# A Protocol for the Comprehensive Flow Cytometric Analysis of Immune Cells in Normal and Inflamed Murine Non-Lymphoid Tissues

**DOI:** 10.1371/journal.pone.0150606

**Published:** 2016-03-03

**Authors:** Yen-Rei A. Yu, Emily G. O’Koren, Danielle F. Hotten, Matthew J. Kan, David Kopin, Erik R. Nelson, Loretta Que, Michael D. Gunn

**Affiliations:** 1 Department of Medicine, Division of Pulmonary and Critical Care Medicine, Duke University Medical Center, Durham, North Carolina, United States of America; 2 Department of Immunology, Duke University Medical Center, Durham, North Carolina, United States of America; 3 Department of Medicine, Division of Cardiology, Duke University Medical Center, Durham, North Carolina, United States of America; 4 School of Medicine, Duke University Medical Center, Durham, North Carolina, United States of America; 5 Department of Molecular & Integrative Physiology, University of Illinois, Champaign, Illinois, United States of America; The University of Melbourne, AUSTRALIA

## Abstract

Flow cytometry is used extensively to examine immune cells in non-lymphoid tissues. However, a method of flow cytometric analysis that is both comprehensive and widely applicable has not been described. We developed a protocol for the flow cytometric analysis of non-lymphoid tissues, including methods of tissue preparation, a 10-fluorochrome panel for cell staining, and a standardized gating strategy, that allows the simultaneous identification and quantification of all major immune cell types in a variety of normal and inflamed non-lymphoid tissues. We demonstrate that our basic protocol minimizes cell loss, reliably distinguishes macrophages from dendritic cells (DC), and identifies all major granulocytic and mononuclear phagocytic cell types. This protocol is able to accurately quantify 11 distinct immune cell types, including T cells, B cells, NK cells, neutrophils, eosinophils, inflammatory monocytes, resident monocytes, alveolar macrophages, resident/interstitial macrophages, CD11b^-^ DC, and CD11b^+^ DC, in normal lung, heart, liver, kidney, intestine, skin, eyes, and mammary gland. We also characterized the expression patterns of several commonly used myeloid and macrophage markers. This basic protocol can be expanded to identify additional cell types such as mast cells, basophils, and plasmacytoid DC, or perform detailed phenotyping of specific cell types. In examining models of primary and metastatic mammary tumors, this protocol allowed the identification of several distinct tumor associated macrophage phenotypes, the appearance of which was highly specific to individual tumor cell lines. This protocol provides a valuable tool to examine immune cell repertoires and follow immune responses in a wide variety of tissues and experimental conditions.

## Introduction

Flow cytometry is used extensively to examine immune cell repertoires and follow immune responses in non-lymphoid tissues. Flow cytometric analysis furnishes important insights into the immune status of a given tissue by providing information about the numbers and phenotypes of the immune cells that the tissue contains. Ideally, a method for performing flow cytometric analysis would be available that is both comprehensive and widely applicable; capable of accurately identifying and quantifying all immune cell types in almost any tissue, either at baseline or during an inflammatory response. Unfortunately, no such method has yet been described. Currently described protocols for the flow cytometric analysis of non-lymphoid tissues represent a compromise between maximizing the number of informative cell surface markers examined and using a limited number of fluorescent channels. For this reason, these protocols display a number of characteristic limitations.

One approach commonly used to examine immune cells in non-lymphoid tissues is to focus on a limited number of cell types. This approach, while often providing important information, does not provide a complete picture of immune cell repertoires and does not allow for examination of dynamic changes in all immune cell populations. A second commonly used approach is to enrich certain rare cell types, such as dendritic cells (DC), by density gradient centrifugation prior to staining and analysis. This limits the number of cell types that can be analyzed and can result in the loss of cells during processing. In addition, some immune cell types have proven to be difficult to clearly identify as distinct populations. This includes several specific members of the granulocyte and mononuclear phagocyte families. Granulocytes include mast cells, eosinophils, basophils, and neutrophils. Although methods for identifying individual granulocyte types have been described, we are not aware of a protocol for the simultaneous identification of all granulocyte types or the incorporation of this into more generalized flow cytometry protocols [1–3].

Mononuclear phagocytes include monocytes, macrophages, and dendritic cells, with each of these being comprised of several subsets [4]. In practice, reliably identifying monocytes, macrophages, and dendritic cells as clearly distinct populations has proven challenging and has spurred significant controversy [5–7]. This is in part due to the fact that some commonly used markers of myeloid populations have proven to be less specific than originally thought. One example of this is CD11c (ITGAX), originally described as a specific marker for dendritic cells, especially when used in conjunction with expression of MHC class II [5]. It is now recognized that several non-DC myeloid populations express CD11c, including alveolar macrophages and resident (Ly6C low) monocytes and that some macrophage populations express both CD11c and MHC class II [8, 9]. Similarly, CD11b has often been used as a marker for all myeloid cell types. However, some myeloid cell subpopulations are negative for CD11b expression, including alveolar macrophage and CD103^+^ dendritic cells, while some non-myeloid cell types, such as mature NK cells, express CD11b [8, 10]. Thus, the commonly used approach of relying on CD11c, CD11b, and MHC class II to distinguish macrophages from DC has proven inadequate [5]. For this reason, there have been efforts in recent years to identify more robust macrophage- or dendritic cell-specific markers.

Markers that have been proposed to distinguish between macrophages and dendritic cells include Mac2, Mac3 (CD107b), CD68, and F4/80 [9, 11–13]. More recently, CD64 and MerTK were shown to be expressed specifically on some non-lymphoid tissue macrophages and are gaining popularity as macrophage-specific markers [14–17]. Use of such markers has improved our ability to identify individual myeloid cells populations. For example, a 10-color panel has been described for use in mouse lungs that reliably distinguishes macrophages from DC and allows identification of 9 distinct myeloid cell populations [8, 9]. While such efforts represent a significant advance, they still do not allow for the comprehensive analysis of all major immune cell populations and have not been validated in other non-lymphoid tissues.

Here, we describe a procedure for the processing, antibody staining, and flow cytometric analysis of murine non-lymphoid tissues that maximizes cell viability, results in minimal cell loss, and allows the simultaneous identification and quantitation of all major leukocyte populations, including the major monocyte, macrophage, dendritic cell, and granulocyte subtypes. This procedure is applicable to all non-lymphoid tissues we have examined, including lung, intestine, heart, liver, kidney, skin, eyes, and mammary gland. This procedure was also found to work well in acutely inflamed tissues, specifically lipopolysaccharide (LPS)-challenged and H1N1 influenza infected pulmonary tissues. Additionally, it allows for the examination of tumor-induced cellular inflammation and the heterogeneity of tumor-associated macrophages (TAMs) across different tumor models.

## Methods

### Mice

Male and female C57BL/6 and FVB mice between 8 and 12 weeks of age were purchased from Charles River Laboratories (Morrisville, NC). MMTV-PyMT mice were initially purchased from Jackson Laboratories and crossed seven generations onto a C57BL/6 background [18, 19]. *Cx3cr1*^*GFP/GFP*^ mice were also purchased from Jackson Laboratories (Bar Harbor, Maine) and cross to C57BL/6 to generate *Cx3cr1*^*wt/GFP*^. All mice were housed at a barrier-free and specific-pathogen-free facility at Duke University School of Medicine (Durham, NC). All procedures were approved by the Institutional Animal Care and Use Committee at Duke University.

### H1N1 Influenza Infection

Male C57Bl/6 mice between 8–10 weeks of age were anesthetized with isoflurane. Animals were treated with 250 PFU of either heat inactivated or active H1N1 influenza virus intranasally, as described previously [20]. Lung tissues were harvested at day 0, 3, and 7 after exposure.

### LPS Challenge

Male C57BL/6 mice between 8–9 weeks of age were anesthetized with 100mg/kg of Ketamine and 10mg/kg of Xylazine. Subsequently, animals were treated intranasally with 12.5μg of LPS derived from Salmonella Enteroca purchased from Sigma Aldrich (St. Louis, MO) in 50ul of PBS. Lung tissues were harvested as described below at 0, 1, 2, 3, and 4 days after exposure.

### Primary and metastatic tumors

Tumors were harvested from MMTV-PyMT mice at ~6 months of age. For primary tumors, 0.2x10^6^ E0771 or 1x10^6^ Met-1 cells were grafted orthotopically into the axial mammary fat pad. Met-1 cells were grafted in 50% matrigel. For the metastasis model, cells 1x10^6^ E0771 cells were injected *i*.*v*. via the tail vein.

### Tissue Processing

#### Lung single cell suspensions

Mice were treated with 50ul of 1000U/ml *s*.*c*. heparin, and then euthanized with Isoflurane. The chest cavity was opened, the left atrium nicked, and the lungs perfused with 10ml of PBS through the right atrium. The trachea was then exposed, a small incision was made at its top, and an 18 gauge angiocath was inserted and secured. Lung was inflated with digestion solution containing 1.5mg/ml of Collagenase A (Roche) and 0.4mg/ml DNaseI (Roche) in HBSS plus 5% fetal bovine serum and 10mM HEPES. Trachea was tied off with 2.0 sutures. The heart and mediastinal tissues were carefully removed and the lung parenchyma placed in 5ml of digestion solution and incubated at 37°C for 30 minutes with gently vortexing every 8–10 minutes. Upon completion of digestion, 25ml of PBS was added; and the samples were vortexed at maximal speed for 30 seconds. The resulting cell suspensions were strained through a 70um cell strainer and treated with ACK RBC lysis solution.

#### Other non-lymphoid organs

Mice were treated with 50ul of 1000U/ml *s*.*c*. heparin, and then euthanized with Isoflurane. After euthanasia, animals were perfused through the left ventricle with PBS. The heart, liver, kidney, small intestine, mammary, skin from the ear, and eyes were collected. All tissues were cut into small strips of ~5mm thickness and placed in digestion solution. Tissues were incubated at 37°C with continuous shaking at 250rpm for 45 minutes and gentle vortexing every 8–10 minutes. For liver and kidney, the above processing resulted in a single cell suspension. For heart, skin, mammary tissues and eyes, gentle teasing with forceps followed by a second round of digestion was required. Upon completion of digestion, 25ml of PBS was added and the samples were vortexed at maximal speed for 30 seconds. The resulting cell suspensions were strained through a 70um cell strainer, pelleted, washed in PBS with 1% BSA, and enumerated with Turk’s solution.

### Flow Cytometry

After cells were counted, and 2x10^6^ cells per sample were stained with Aqua Live/Dead viability dye (Life Technologies) according the manufacturer’s instructions. Cells were then incubated in blocking solution containing 5% normal mouse serum, 5% normal rat serum, and 1% FcBlock (eBiosciences, San Diego, CA) in PBS and then stained with a standard panel of immunophenotyping antibodies (See S2 Table for a list of antibodies, clones, fluorochromes, manufacturers, and concentrations) for 30 minutes at room temperature. After staining, cells were washed and fixed with 0.4% paraformaldehyde in PBS. Data was acquired with a BD LSRII flow cytometer using BD FACSDiva software (BD Bioscience). Compensation was performed on the BD LSRII flow cytometer at the beginning of each experiment. Data were analyzed using Flowjo v10. Cell sorting for cytospins was performed on a BD Aria II. The collected cells were stained with a Jenner-Giemsa Stain Kit (ENG Scientific Inc, Clifton, NJ) and examined by light microscopy.

### Statistical Analysis

Significant differences between groups were determined using unpaired t tests. All analyses were performed using Excel and GraphPad Prism, version 6.00 (GraphPad Software, San Diego, CA). Data are shown as means ± SEMs. P<0.05 was considered statistically significant.

## Results

### Immunophenotyping of leukocyte subsets in murine lung tissue

We developed a protocol for the preparation and flow cytometric analysis of non-lymphoid tissue immune cells that: 1) minimizes the loss of cells during tissue preparation; 2) allows for the identification of macrophages and DC as clearly distinct populations; and 3) allows the identification of myeloid cell subsets, such as interstitial macrophages, which have previously been difficult to identify as discrete populations. This protocol was initially developed for mouse lung tissues, and then adapted to other tissues. In this section we present an overview of the protocol as it applies to mouse lungs.

In initial studies, we found that substantial cell loss and/or death occurs when tissues are subjected to the common practice of mechanical tissue dissociation and density gradient enrichment (data not shown). To avoid mechanical tissue dissociation in our protocol, lungs are inflated via tracheal infusion with a collagenase/DNase containing solution, incubated, then subjected to gentle agitation. Relative to treatment with proteolytic enzymes and mincing, this procedure results in a marked increase in the viable cell yield in the resulting single cell suspension on a per lung basis (4.33x 10^7^ ± 1.87x10^6^ vs. 8.11x10^6^ ± 9.30x10^5^, p<0.0001), especially within the monocyte and tissue macrophage subsets (data not shown). To eliminate subsequent cell loss, the single cell suspension is not subjected to density gradient enrichment but instead undergoes immediate staining with fluorochrome-conjugated antibodies.

**Fig 1:**
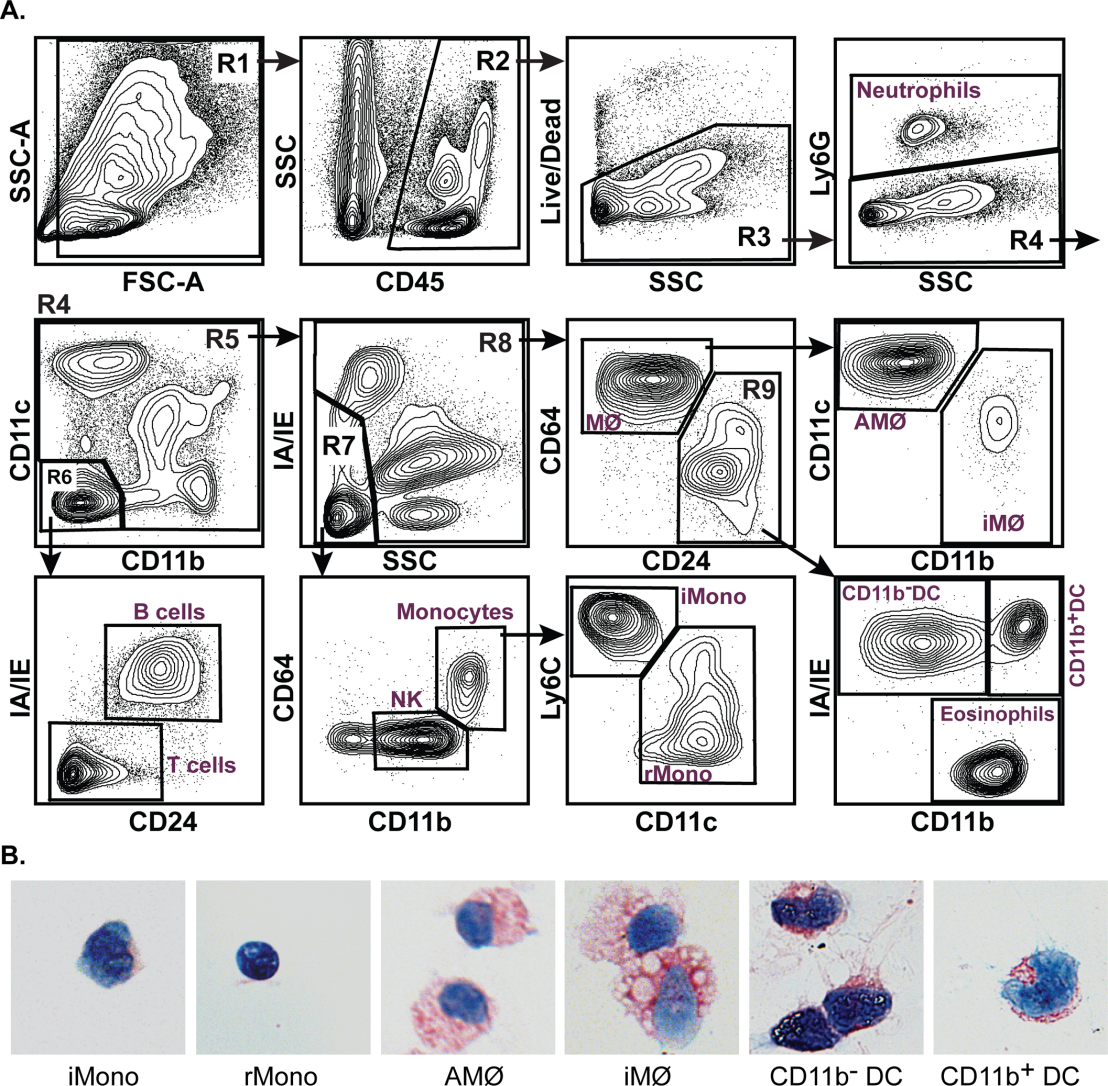
Flow cytometric analysis of mouse lung. **A.** Contour plots of windows and gating strategy used for the identification of major immune cell populations in normal mouse lungs. Gates containing multiple cell populations are numbered (R1-R9). Gates containing a single cell population are labeled with the included cell type. These include: T cells, B cells, NK cells, neutrophils, eosinophils, inflammatory monocytes (iMono), resident monocytes (rMono), alveolar macrophages (AMФ), interstitial macrophages (iMФ), CD11b^-^ dendritic cells (CD11b^-^ DC), and CD11b^+^ dendritic cells (CD11b^+^ DC). The distribution of cells within all panels is representative of 20 independent experiments. **B.** Photomicrographs of Jenner-Giemsa stained individual mononuclear phagocyte cell types purified by FACS using the gating strategy shown in A.

The gating and cell identification strategy for our basic staining protocol is shown in Fig 1 and is as follows: After cell doublets and clumps are eliminated by FSC-H vs. FSC-A gating (not shown) and debris is eliminated using FSC-A vs. SSC-A (R1), leukocytes are identified based on expression of CD45 (R2). Total live leukocytes (R3) are then identified based on the exclusion of a Live/Dead dye. Typically, we find that approximately 50% of total lung cells are live CD45^+^ leukocytes. Neutrophils are distinguished from all other leukocytes (R4) based on their expression of the neutrophil-specific marker, Ly6G. Positive staining for either CD11b or CD11c on non-neutrophils is then used to distinguish the remaining myeloid leukocytes (R5) from CD11b^-^CD11c^-^ lymphoid cells (R6). This specific gating does not account for the small population of lymphocytes that are CD11c^+^, but these will be identified in subsequent gating. The CD11b^-^ CD11c^-^ lymphoid cells can be further differentiated into MHC class II (IA/IE)^+^ CD24^+^ B cells and IA/IE^-^ CD24^-^ T cells. The identity of T and B cells was confirmed by their expression of B220, and CD3, respectively (S1B Fig).

The myeloid leukocytes (R5) are first subdivided based on their pattern of side scatter vs. MHC class II expression. The IA/IE^-^ SCC^lo^ population (R7) contains a small population of CD11c^+^ lymphocytes, NK cells, and monocytes. These can be distinguished based on their expression of CD11b and CD64: lymphocytes are CD11b^-^ CD64^-^, NK cells are CD11b^int^ CD64^-^, and monocytes are CD11b^hi^ CD64^int^. The monocytes can be further subdivided into Ly6C^hi^ CD11c^-^ inflammatory monocytes and Ly6C^-^ CD11c^+^ resident monocytes. The IA/IE^+^ or SCC^hi^ cells within R5 (R8) consist of macrophages, DC, and eosinophils. To distinguish macrophages from DC and eosinophils, we relied on the expression of CD24 and CD64 rather than more commonly used markers, such as CD11c or MHC class II. Using this strategy, CD64^+^ CD24^-^ macrophages (MФ) can be clearly distinguished from the CD64^-^ CD24^+^ DC and eosinophils (R9). Within this R9 gate, 3 cell populations can be distinguished based on their expression of MHC class II and CD11b: IA/IE^-^ CD11b^+^ eosinophils, IA/IE^+^ CD11b^-^ DC, which correspond to CD103^+^ CD11b^-^ DC, and IA/IE^+^ CD11b^+^ DC, which correspond to CD103^-^ CD11b^+^ DC (S1C and S7 Figs).

In normal lungs, cells with the CD64^+^ MФ gate can be subdivided into CD11b^-^ CD11c^+^ alveolar macrophages (AMФ) and CD11b^+^ CD11c^-^ interstitial macrophages (iMФ) using antibodies already present in the panel. However, because of the large diversity of macrophage phenotypes seen in different organs and different inflammatory conditions, the specific staining strategies used to identify macrophage subsets need to be modified on a case-by-case basis. In some cases, this will require the use of additional Abs. In the sections below, we will provide examples of the macrophage staining strategies we have found to work best for specific tissues and conditions. The Ab staining and gating strategy shown above was validated by purifying individual myeloid cell types by FACS and examining the purified cells by Jenner-Giemsa staining. In all cases, the purified cells display their expected morphology (Fig 1B).

The basic staining protocol outlined above identifies all major immune cell types in most tissues. It does not, however, specifically identify three cell types: mast cells, basophils, and plasmacytoid DC (pDC). We have found that, in most tissues under most conditions, the frequency of these cell types is so small (<0.1% of CD45^+^ cells) that they can be ignored without significantly affecting the accuracy of quantitation of the major cell types. However, in tissues or conditions in which the frequency of one or more of these cell types is increased or needs to be specifically identified, this can be accomplished by the examination of one or more additional cell surface markers. For example, tissue mast cells and basophils can be identified by the addition of antibodies against the high-affinity IgE receptor, FcεRI, and CD117 (cKit) with only a small adjustment in gating (S1A Fig). Similarly, pDC can be identified by the inclusion of anti-mPDCA-1 antibody (data not shown).

### Standard Method for Immunophenotyping non-lymphoid tissues

Once optimized for the immunophenotyping of murine lung tissues, we applied our procedure to the analysis of a wide range of non-lymphoid tissues, including heart, liver, small intestine, kidney, skin, mammary glands, and eyes. To generate single cell suspensions, we adapted our tissue processing method for use in denser organs. Tissues were cut into small strips, placed in collagenase/DNase solutions, and continuously agitated at 37°C. Most tissues dissolved into single cell suspensions with gentle agitation alone. A few tissues, including heart, skin, and eyes, required gentle teasing with forceps followed by a second round of digestion with agitation. A high level of cell viability was maintained with this strategy (data not shown). The resulting single cell suspensions were stained with our antibody panel and analyzed as described above.

**Fig 2:**
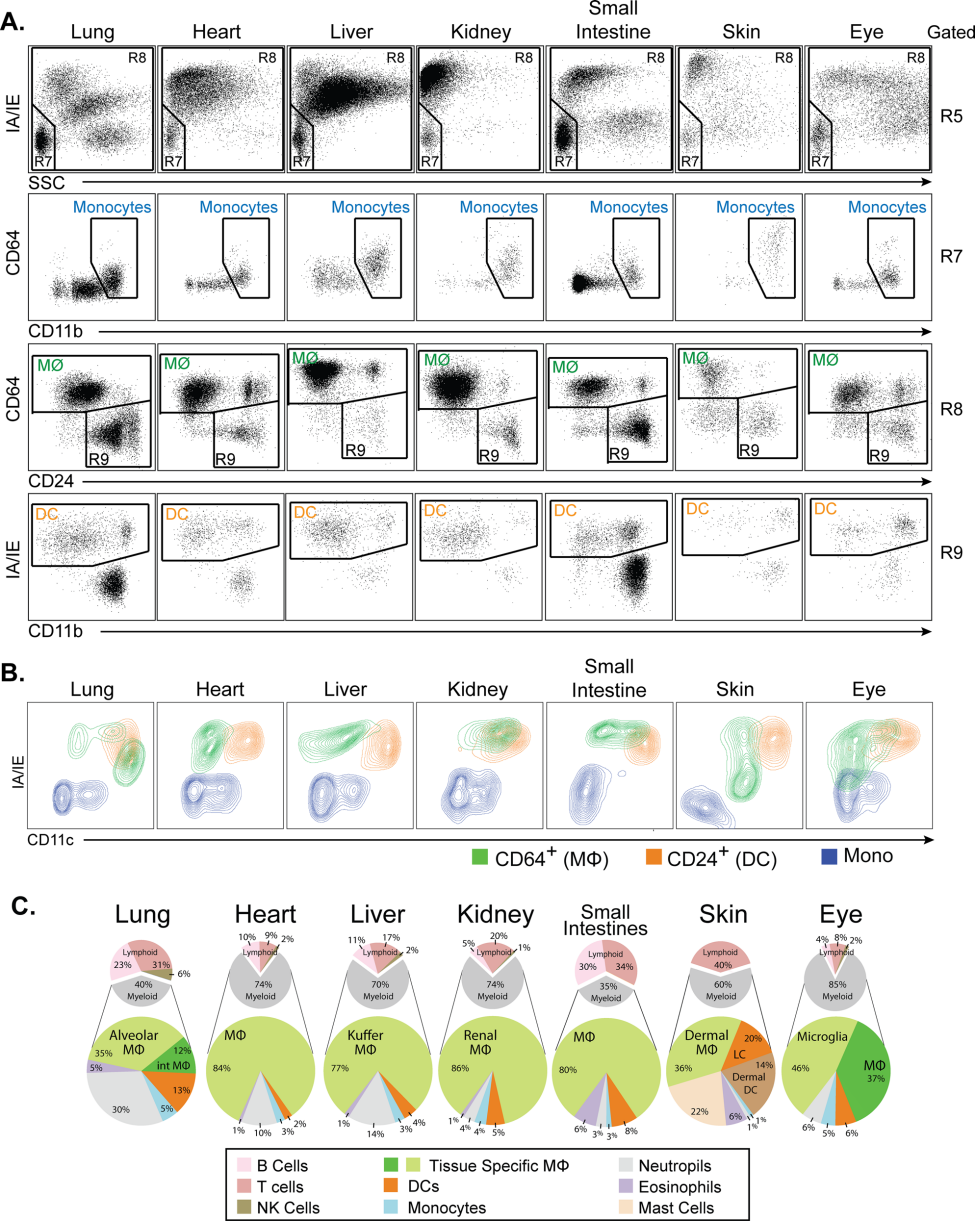
Flow cytometric analysis of other non-lymphoid organs. **A.** Dot plots showing selected windows and gating strategy as applied to the identification of major immune cell populations in the indicated tissues. The gating strategy begins on the top row, which is gated on R5 from Fig 1. Selected populations specifically identified and color-coded include monocytes (blue), macrophages (MФ, green), and dendritic cells (DC, orange). Populations not labeled conform to those shown in Fig 1. Figures are representative of 3 independent experiments. **B.** Contour plots of CD11c vs. MHC class II expression for monocytes (blue), macrophages (green), and dendritic cells (orange) from gates shown in panel A. **C.** Pie charts showing the relative frequencies of all major immune cell types in the indicated tissues. The upper small charts display cell type frequencies as a percentage of total tissue of CD45^+^ cells. The lower large charts display cell type frequencies as a percentage of total tissue of myeloid cells. Percentage represents the means of values obtained in 3 independent experiments.

As in lung, our basic staining protocol and gating strategy clearly identified total live leukocytes, neutrophils, B cells, and T cells (data not shown). As shown in Fig 2A, this protocol was also able to clearly distinguish monocytes, macrophages, DC, NK cells, and eosinophils in all tissues examined. Examination of SSC vs. MHC class II expression allowed the separation of NK cells and monocytes (R7) from mature myeloid cells (R8) as distinct populations in almost all tissues (Fig 2A, top row). Examination of multiple tissues also led to a minor adjustment in the gate used to define macrophages based on CD64 vs CD24 expression (Fig 2A, third row). In several tissues, a population of CD64^+^ CD24^+^ cells was present that had not appeared in lungs. In examining this population, we find that it exhibits identical FSC/SCC characteristics and a pattern of macrophage marker expression that is identical to CD64^+^ CD24^-^ macrophages (data not shown). Based on these findings, we classify CD64^+^CD24^+^ cells as macrophages. Based on this revised gating, examination of CD64 vs CD24 expression clearly separated macrophages (MФ) from DC and eosinophils (R9) in all tissues examined (Fig 2A, third row). In most tissues, this gating accounted for >98% of the mature myeloid cells identified in the R8 gate. The remaining CD64^-^ CD24^-^ cells were found to represent small numbers of lymphocytes or monocytes that had not been captured in the R6 or R7 gates. If required, these cells can be specifically gated on and individual populations quantified by the examination of other cell surface markers. The one exception to this is in skin, where a significant population of CD64^-^ CD24^-^ cells is present. These represent dermal dendritic cells, which have been previously described as being CD24^-^ [21].

The above findings suggest that the use of CD64 vs. CD24 expression to distinguish macrophages from dendritic cells is superior to the commonly used method of using CD11c vs. MHC class II expression to distinguish these cell types. To examine this point specifically, we plotted the positions of monocytes (blue), macrophages (green), and DC (orange) from various tissues based on their expression of CD11c and MHC class II (Fig 2B). As this plot demonstrates, there is substantial overlap of macrophage and DC populations when examined by CD11c vs. MHC class II expression. In the kidney, macrophages and DC are indistinguishable using this method, while in the eye, macrophages display considerable overlap with monocytes. This confirms prior observations that CD11c and MHC class II expression are inadequate for the discrimination of macrophages from dendritic cells and monocytes.

### Defining the composition of all immune cells in non-lymphoid tissues

Our overall goal was to develop a method for performing flow cytometric analysis that is capable of accurately identifying and quantifying all immune cell types in almost any non-lymphoid tissue. To demonstrate the applicability of the above procedure in simultaneously identifying and quantifying all immune cell types in almost any non-lymphoid tissue, we quantified these cell types in seven different non-lymphoid tissues (Fig 2C). We find that the percentage of CD45^+^ leukocytes varies widely across non-lymphoid tissues (2% to 49%) but is consistent within tissues (data not shown). In organs that contain mucosal-associated lymphoid tissues (i.e. lung and small intestine), the majority of leukocytes are lymphoid cells (65%-74%) (Fig 2C). In contrast, non-mucosal tissues (i.e. heart, liver, and kidney) contain predominately myeloid cells (59%-71%). Within the myeloid compartment, tissue macrophages are the most abundant cell type in non-lymphoid organs, followed by granulocytes, DC, and finally monocytes. The exception to this is skin, where DC of various subtypes predominate. We should note that the using the basic panel, the identification of Langerhans cells and various subsets of dermal DC are possible. But, adding additional markers for confirmation is greatly encouraged. Langerhans cells being defined as CD207^+^ CD172a^+^ F4/80^+^ EpCAM^+^ CD11b^Int^ CD24^hi^ (data not shown). Overall, the procedure we describe allows the determination of immune cell composition in a broad spectrum of non-lymphoid tissues.

### Comparison of CD64/CD24 expression with other myeloid markers

**Fig 3:**
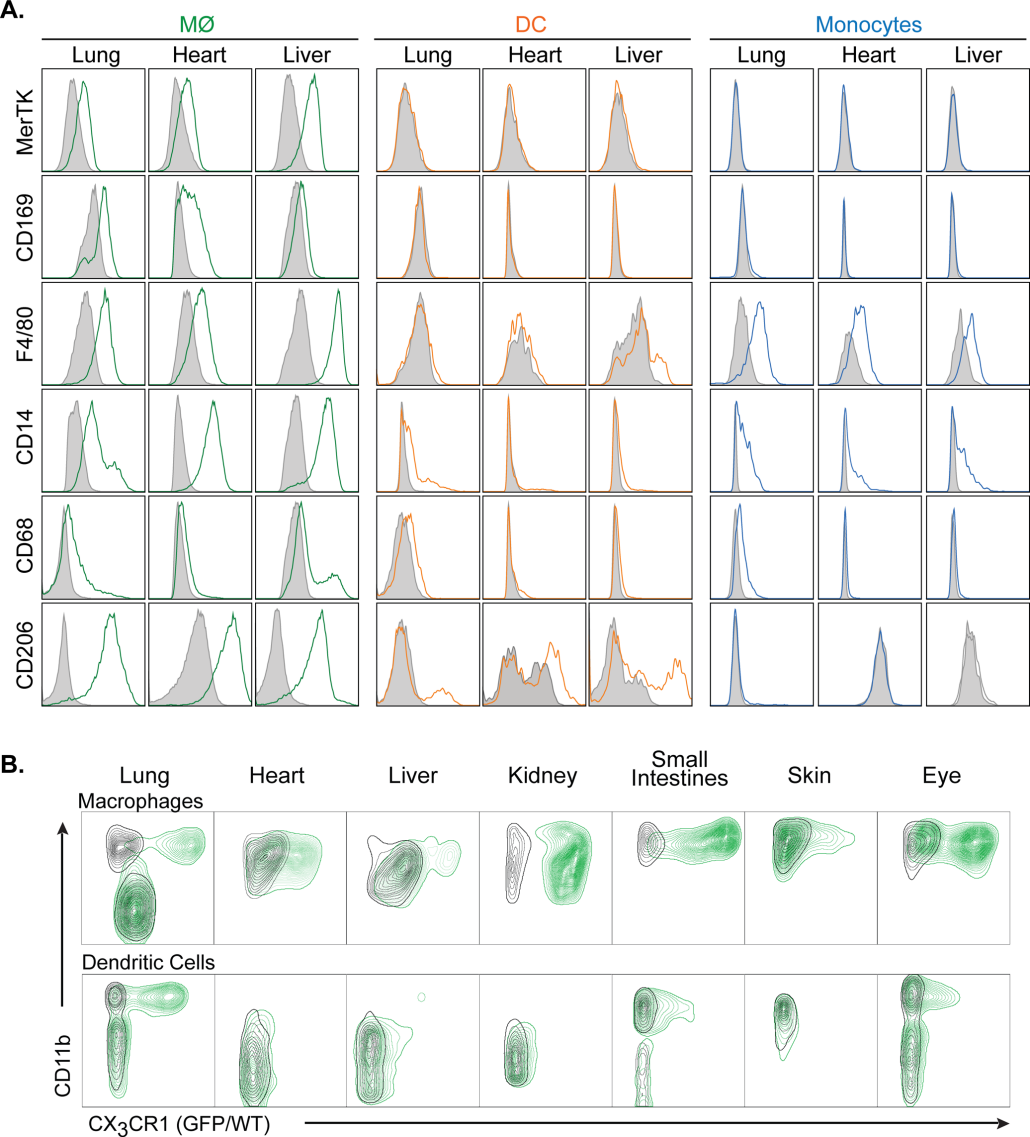
Expression of common myeloid markers on specific cell populations in non-lymphoid tissues. A. Histograms displaying the expression levels of MerTK, CD169, F4/80, CD14, CD68, and CD206 on macrophages (MФ), dendritic cells (DC), and monocytes in the indicted tissues. Filled grey plots represent isotype controls. All panels are representative of 3 independent experiments. **B.** Contour plots of CD11b vs. Green Fluorescent Protein expression in macrophages (upper row) and dendritic cells (lower row) in the indicated tissues. Each panel shows cells obtained from wild type mice (black) overlaid with cells obtained from *Cx3cr1*^*wt/GFP*^ knock-in mice (green). All panels are representative of 3 independent experiments.

To confirm that the cell staining and gating strategy we developed can accurately distinguishes macrophages from DC, we examined the expression of known macrophage-specific cell surface markers on the macrophage, DC, and monocyte populations defined in Fig 2A. Based on gene expression analysis, Gautier et al identified MerTK and CD169 as being highly specific macrophage markers in multiple tissues [16]. We find that both MerTK and CD169 are expressed on macrophages, but not monocytes or DC, in all tissues examined (Fig 3A and S1 Table). F4/80 is a widely used marker for monocytes and tissue macrophages. Consistent with this, we find F4/80 to be expressed on all monocytes and macrophages in the tissues we examined. F4/80 is also expressed on small subsets of DC and eosinophils (data not shown). CD14 expression has been described on monocytes macrophages, some DC types, and, at a lower level, on neutrophils [22]. We find that CD14 is expressed at high levels on macrophages, at lower and more variable levels on monocytes, and on a subset of DC. CD206, the mannose receptor, is expressed on macrophages and DC and has been used as a marker for M2 macrophage polarization [23]. We find that CD206 is expressed at high levels by all tissue macrophage populations and by a subset of DC in some tissues.

Increasingly, the expression of CX3CR1, the fractalkine receptor, is used to identify myeloid cells by identifying GFP^+^ cells in *Cx3cr1*^*wt/GFP*^ mice [24]. In these mice, GFP^+^ cell populations have been described to include monocytes, some macrophage subsets, and some DC subsets [24]. To examine the specific cell populations, as identified in our basic staining protocol, that express CX3CR1, we subjected *Cx3cr1*^*wt/GFP*^ mice to flow cytometric analysis and examined GFP expression in monocytes, macrophages and DC. As expected, monocytes in all tissues display significant expression of GFP (data not shown). As shown in Fig 3B, panels were gated either on CD64^+^CD24^-^ macrophages (MФ) or on CD11c^+^ cells in gate R9 (DC) as in Fig 1. Contour plots are shown for macrophages and DC obtained from WT (black) and *Cx3cr1*^*wt/GFP*^ (green) mice. We find that expression of *Cx3cr1*-GFP in macrophages varies significantly by tissue (Fig 3B, upper row). In lungs, *Cx3cr1*-GFP expression clearly distinguishes CD11b^-^ alveolar macrophages from CD11b^+^ interstitial macrophages. In small intestines and kidneys, almost all macrophages express GFP, while in liver and skin, most macrophages are GFP^-^. In the heart, a portion of macrophages expresses *Cx3cr1*-GFP. In eyes, two distinct populations appear: CX3CR1^-^ extra-retinal macrophages and CX3CR1^hi^ retinal microglia. In all tissues examined, CD11b^-^ DC are uniformly negative for *Cx3cr1*-GFP expression (Fig 3B, lower row). In addition, subsets of CD11b^+^ DC also express GFP in those tissues where they are present.

### Identification of myeloid cell populations in acute inflammation

**Fig 4:**
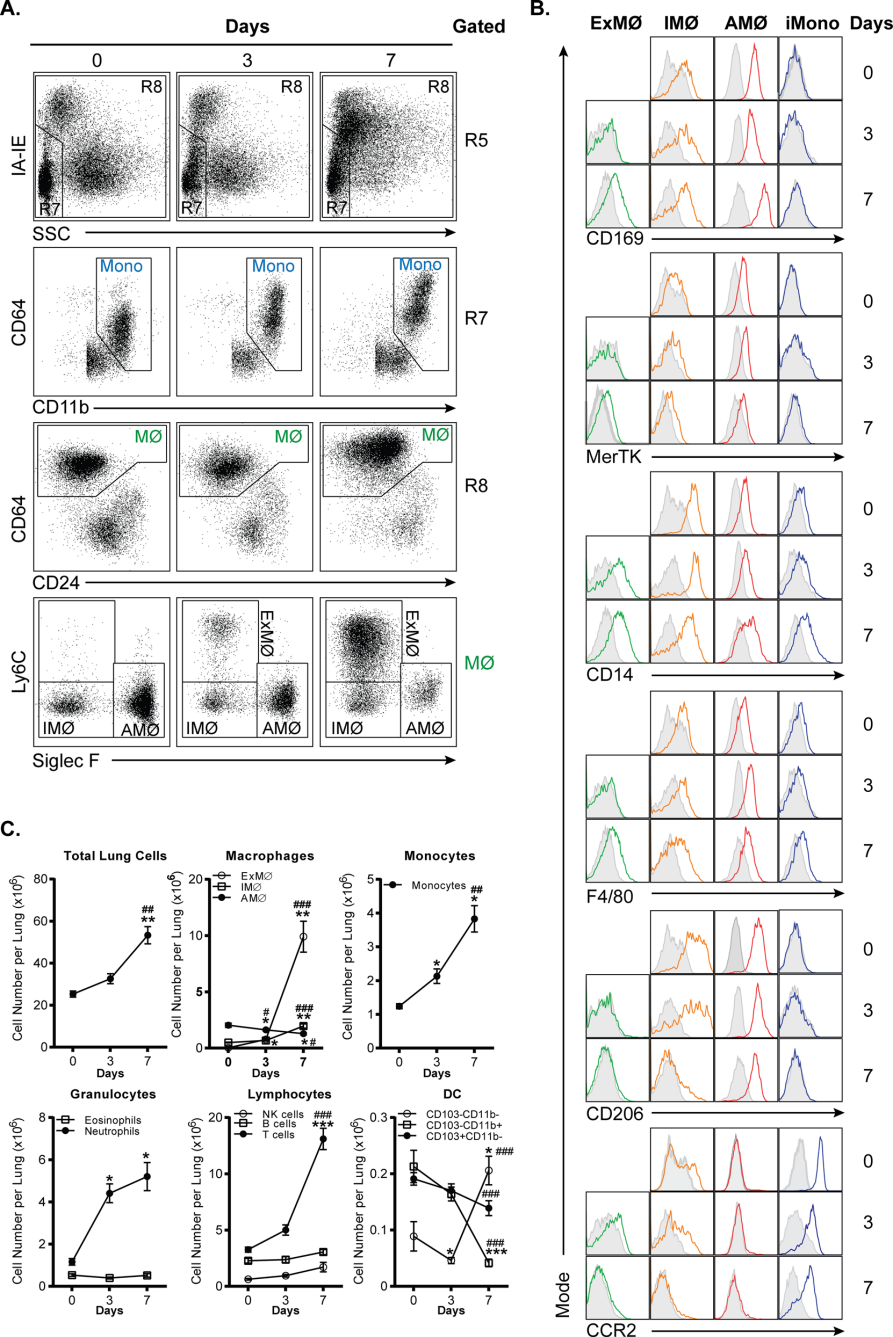
Flow cytometric analysis of H1N1 influenza infected lung tissues. **A.** Dot plots showing gating strategy used to identify monocytes and macrophage (MФ) subpopulations in H1N1 influenza infected lung tissues. The gating strategy begins on the top row, which is gated on R5 from Fig 1. **B.** Histograms depicting CD169, MerTK, CD14, F4/80, CD206, and CCR2 expression on interstitial macrophages (IMФ), exudative macrophages (ExMФ), alveolar macrophages (AMФ), and Ly6C^high^ inflammatory monocytes (iMono). C. Line graph depicting immune cell profiles on day 0, 3, and 7 after H1N1 influenza exposure. And, n = 3 for day 0; n = 9 for day 3; and n = 7 for day 7. *p<0.05, **p<0.01, and ***p<0.001, compared to day 0. #p<0.05, ##p<0.01, and ###p<0.001, compared to day 3.

The above data demonstrates the use of our staining protocol and gating strategy in normal tissues. To determine if our protocol is applicable to the analysis of acutely inflamed tissues, we examined immune cell populations in lungs after H1N1 influenza infection or intranasal LPS administration. A representative gating of H1N1 influenza infection is shown in S2 Fig. The results for H1N1 influenza infected lung tissues are shown in Fig 4A. Similar to the gating of normal lung tissues described in Fig 1A, we separate monocytes (R7) from macrophages (R8) based on SSC and IA-IE expression. Note that monocytes and macrophages do not appear as fully distinct populations for reasons discussed below. Within the R7 gate, we separate NK cells from total monocytes based on CD11b vs CD64 expression. Within the R8 gate, CD64 vs. CD24 clearly distinguishes macrophages from dendritic cells.

In the bottom row of Fig 4A, we have altered our strategy for examining macrophage subsets to account for the presence of a macrophage population that is not seen in normal lungs. During influenza infection, large numbers of inflammatory monocytes are recruited to the lung, where they differentiate into inflammatory monocyte-derived macrophages, also referred to as exudative macrophages (ExMФ) [25–28]. By examining Siglec-F vs Ly6C expression, we can distinguish three macrophage populations: AMФ (Siglec-F^+^ Ly6C^-^), iMФ (Siglec-F^-^ Ly6C^-^), and the above noted ExMФ (Siglec-F^-^ Ly6C^+^), which are not present at day 0. As shown in Fig 4B, over the course of infection, ExMФ upregulate the tissue macrophage-specific markers, CD169 and MerTK and also exhibit increased expression of macrophage-associated markers such as CD14 and F4/80. CD206 expression was not detected on these cells at any time point. ExMФ also downregulate CCR2 expression as they mature from inflammatory monocytes. As we have previously described, the transition from monocyte to ExMФ occurs gradually, resulting in large numbers of cells in the lung that display a phenotype intermediate between monocytes and macrophages [29]. It is for this reason that a clear distinction between these cell types cannot be made during influenza infection, necessitating a classification that is somewhat arbitrary. For the sake of simplicity, here we chose to use the same criteria that we use for monocytes and macrophages at baseline. Despite this ambiguity, our gating strategy clearly distinguishes ExMФ from iMФ and AMФ.

One of the advantages of this standard protocol and gating strategy is its capacity to track dynamic changes in multiple immune cell populations simultaneously. This is illustrated in Fig 4C, where immune cells present in influenza infected lung tissues were quantified as the percentage of CD45^+^ cells and as absolute cell numbers (S3D Fig). As expected, neutrophil frequency peaks around day 3 and rapidly declines thereafter. After day 3, ExMФ and T cells begin to accumulate. There is a decline in the frequency AMФ, DC, and B cells over the seven days examined. IMФ and NK cell frequencies do not change significantly.

**Fig 5:**
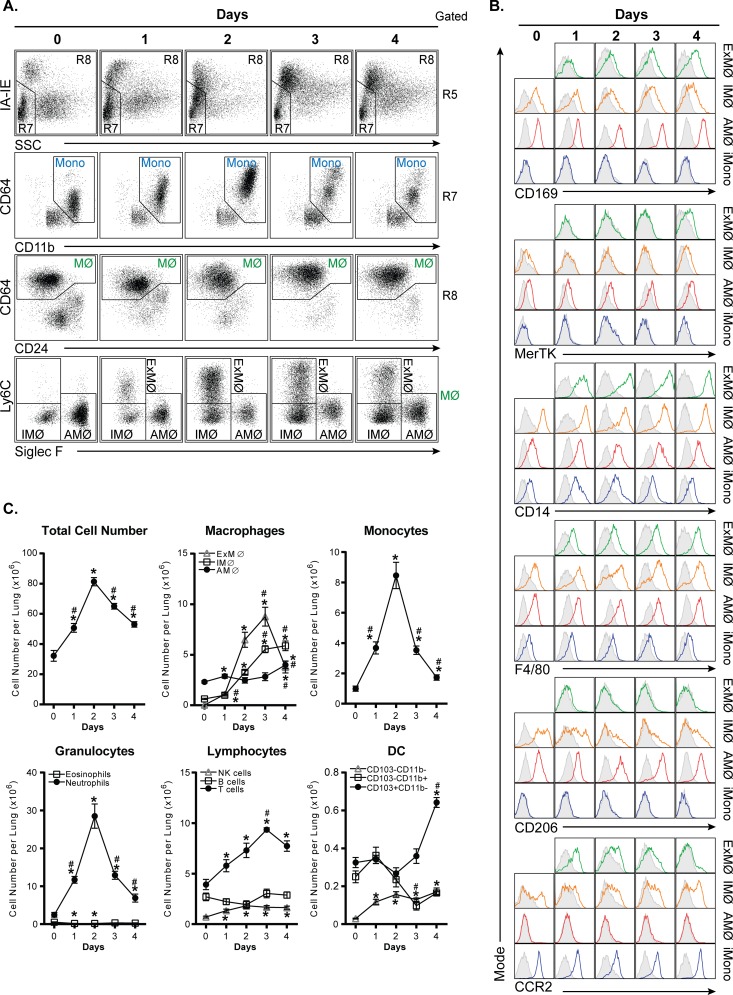
Flow cytometric analysis of LPS exposed lung tissues. **A.** Dot plots showing gating strategy used to identify monocytes and macrophage (MФ) subpopulations in intranasal LPS exposed lung tissues. The gating strategy begins on the top row, which is gated on R5 from Fig 1. **B.** Histograms depicting CD169, MerTK, CD14, F4/80, CD206, and CCR2 expression on interstitial macrophages (IMФ), exudative macrophages (ExMФ), alveolar macrophages (AMФ), and Ly6C^high^ inflammatory monocytes (iMono). C. Line graphs depicting immune cell profiles on 0, 1, 2, 3, and 4 days after LPS exposure. And, n = 3 for day 0; n = 6 for day 1 and 4; n = 5 for day 2 and 3. *p<0.05, **p<0.01, and ***p<0.001, compared to day 0.

To further validate that our protocol is applicable to acute inflammatory conditions, we examined immune cell profiles in the well-characterized LPS-induced pulmonary inflammation model. Fig 5A shows the gating of LPS-exposed lungs from day 0–4. Monocyte and macrophages were identified using the gating strategy described in Figs 1A and 4A. Similar to influenza infection, a new population of Siglec-F^-^ Ly6C^+^ ExMФ arises after LPS exposure (Fig 5A, bottom row). Over the 4 days after LPS exposure, these macrophages increase expression of the tissue macrophage-specific markers, CD169 and MerTK, and macrophage-associated markers, CD14 and F4/80 (Fig 5B). They also do not express CD206 and down-regulate CCR2 expression upon maturation (Fig 5B). However, LPS exposure induces a more accelerated inflammatory response, relative to influenza. Monocyte accumulation peaks by day 2 after LPS exposure and ExMФ frequency peaks by day 3. After day 3, AMФ and DC frequencies begin to recover (Fig 5C and S4D Fig). Thus, the protocol we describe is applicable to tracking cell populations in acutely inflamed lung tissues.

### Identification of macrophage subpopulations in primary mammary tumors

**Fig 6:**
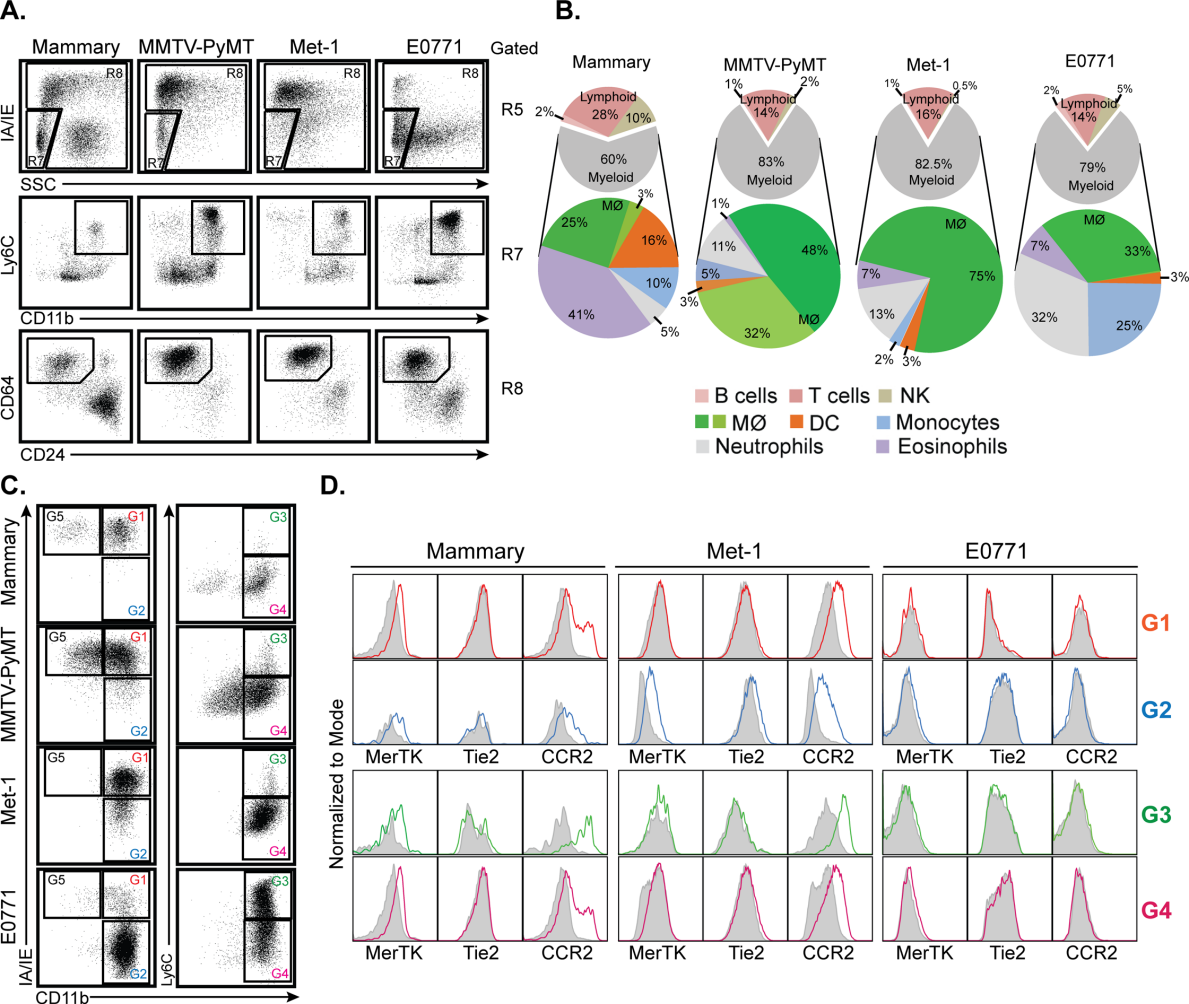
Flow cytometric analysis of primary mammary tumors. **A.** Dot plots showing gating strategy used to identify macrophages (MФ) and inflammatory monocytes in normal mammary tissues and MMTV-PyMT, Met-1, and E0771 mammary tumors. The gating strategy begins on the top row, which is gated on R5 from Fig 1. **B.** Pie charts showing the relative frequencies of major immune cell types in normal mammary tissues and mammary tumors formatted as in Fig 2C. n = 2 for MMTV-PyMT tumors, and n = 3 for all other tissues. **C.** Dot plots of CD11b vs. MHC class II and CD11b vs. Ly6C expression for normal tissue and tumor-associated macrophages. All panels gated on the MФ gate in panel A. **D.** Histograms of MerTK, Tie2, and CCR2 expression on the normal tissue and tumor-associated macrophage subpopulations identified in panel C. Filled grey plots represent isotype controls. All panels are representative of 3 independent experiments.

To determine if our protocol is applicable to the analysis of more chronic inflammation in non-lymphoid organs, we examined immune cell populations in mammary tumors. We chose this model because mouse mammary tumor models have been particularly well characterized and tumors are known to contain heterogeneous populations of myeloid cells that have, in some cases, been difficult to characterize [30, 31]. We examined the immune cell composition of normal mammary tissues, MMTV-PyMT spontaneous mammary tumors, and mammary tissues implanted with the Met-1 or E0771 breast cancer cell lines [18]. The complete gating strategies for normal mammary tissue and MMTV-PyMT tumors are shown on S5 and S6 Figs. The results of gating on myeloid cells from these tissues are shown in Fig 6A. Here, we have displayed monocytes and NK cells from gate R7 in CD11b vs. Ly6C plots to highlight the presence of inflammatory monocytes (Fig 6A, middle row). As in normal tissues, examination of SSC vs. MHC class II expression allowed the separation of NK cells and monocytes (R7) from mature myeloid cells (R8), while CD64 vs CD24 expression clearly separated macrophages (MФ) from DC and eosinophils (R9) in tumors (Fig 6A, third row).

Quantitation of all immune cell populations in normal mammary tissues and tumors demonstrates that macrophages, inflammatory monocytes, and neutrophils display the most marked accumulation in tumors, but that the pattern of cell accumulation is highly tumor-specific, even with Met-1 cells originating from a MMTV-PyMT tumor (Fig 6B). CD45^+^ leukocytes represent 17–27% of cells within the tumor digests (data not shown). All tumors contain of similar percentages of lymphocytes with 15–17% of immune cells being T cells and 1–2% B cells. In both the MMTV-PyMT and Met-1 models, macrophages represent the vast majority (80% and 75%) of tumor immune cells. In contrast, E0771 tumors display only a small relative increase in macrophages, but a marked accumulation of inflammatory monocytes and neutrophils.

Tumor associated macrophages (TAMs) are recognized as being highly heterogeneous, with multiple specific phenotypes previously described [30]. To determine the extent to which our basic staining panel could be used to identify specific subtypes of TAMs, we examined the expression of our other staining panel markers on the CD64^+^ CD24^-^ macrophage population in each tumor. We found that MHC class II, CD11b, and Ly6C displayed the most heterogeneous expression on TAMs and allowed the identification of multiple distinct TAM subsets (Fig 6C). In normal mammary tissues, CD64^+^ CD24^-^ cells include a predominant CD11b^+^ IA/IE^+^ Ly6C^-^ population and a minor CD11b^-^ IA/IE^+^ Ly6C^-^ population. Both these populations increase substantially in MMTV-PyMT tumors, while in Met-1 tumors almost all TAMs display the CD11b^+^ IA/IE^+^ Ly6C^-^ phenotype. In marked contrast, almost all the TAMs in E0771 tumors are CD11b^+^ IA/IE^-^ and the majority of these cells express Ly6C at high levels.

TAMs in MMTV-PyMT tumors have previously been characterized in detail [30]. To further characterize the TAM subsets in Met-1 and E0771 tumors, we examined their expression of MerTK, Tie2, and CCR2 (Fig 6D). Interestingly, we find that MerTK is not expressed on most TAM subsets in Met-1 or E0771 tumors. At present the physiologic significance of this is unknown. Tie2 expression on TAMs has been associated with a pro-angiogenic macrophage activity [32]. We find a low but significant level of Tie2 expression on only one TAM population, the minor CD11b^+^ IA/IE^-^ subset of TAMs seen in Met-1 tumors. Finally, the low expression of MHC class II and the high expression of Ly6C on E0771 tumor TAMs suggested that these cells may be relatively immature macrophages that still display some characteristics of inflammatory monocytes [30]. However, these cells do not express CCR2, the chemokine receptor used by inflammatory monocytes to enter tissues. In contrast, all macrophage subtypes in Met-1 tumors express CCR2, which is also seen on a portion of macrophages in normal mammary tissue. This finding suggests that E0771 tumor TAMs are not just immature, but that the E0771 tumor environment induces specific phenotypic changes in macrophages. A comparison of these cells with MMTV-PyMT and Met-1 tumor TAMS suggest that TAM phenotypes are not determined by a generalized anti-tumor response, but are specifically determined by individual highly tumor-specific factors.

### Comparative analyses of TAM Populations in Primary Site and Lungs

**Fig 7:**
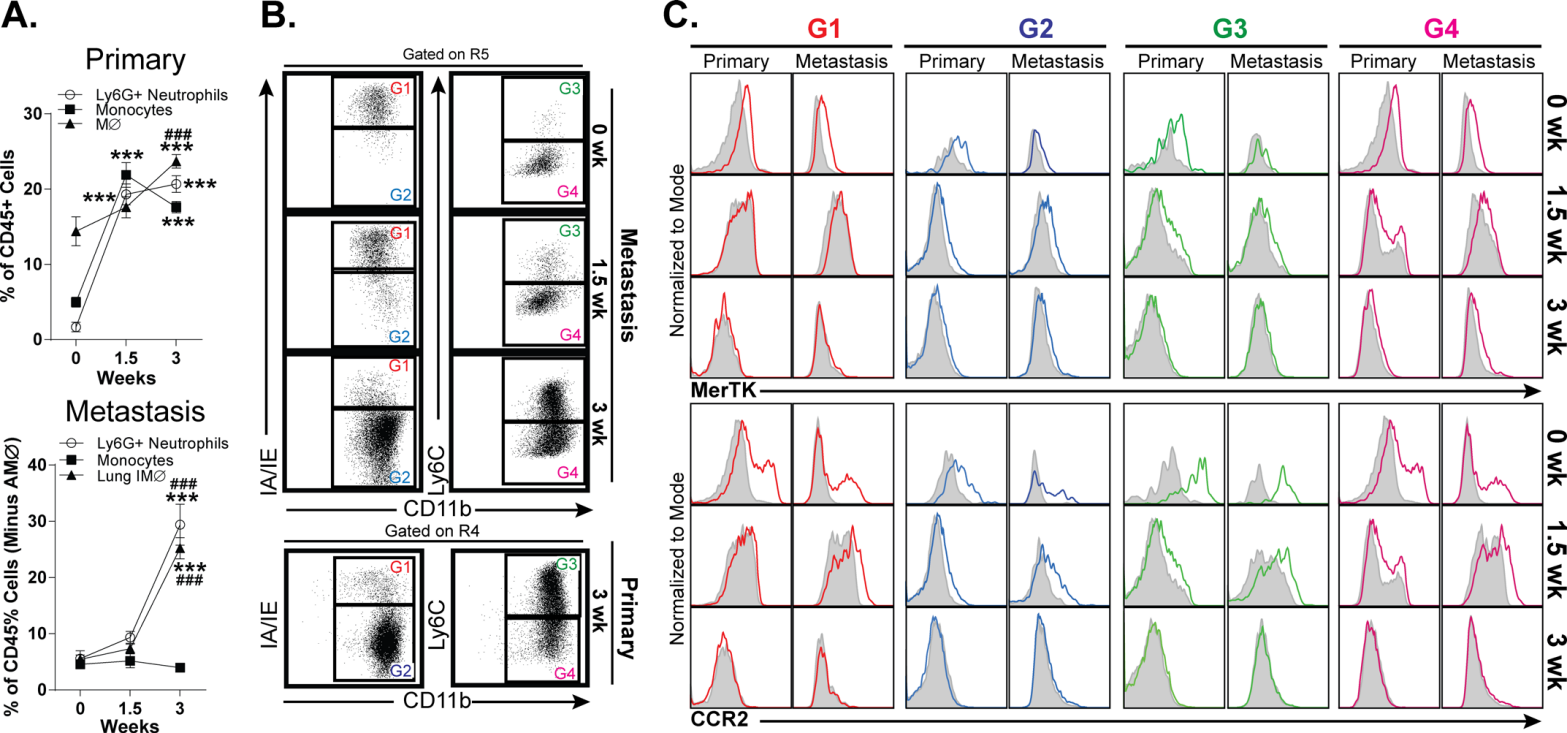
Comparison of myeloid cell accumulation and tumor associated macrophage phenotypes in model E0771 primary tumors and metastases. **A.** Time course of neutrophil, monocyte, and macrophage accumulation in model E0771 primary tumors and metastases over a 3-week time course. n = 3, ***p<0.001 (compared to 0 weeks). ###p<0.001 (compared to 1.5 weeks). **B.** Dot plots of CD11b vs. MHC class II and CD11b vs. Ly6C expression on tumor-associated macrophages in model E0771 primary tumors and metastases. **C.** Histograms of MerTK and CCR2 expression on the tumor-associated macrophage subpopulations identified in panel B. Filled grey plots represent isotype controls. All panels are representative of 3 independent experiments.

The finding that TAM phenotypes are highly tumor-specific raises the question of whether this is purely an effect of the tumor or if there are also tissue-specific effects, as in the case of metastases. To address this question, we compared the immune cell infiltrates in a model of primary tumors (E0771 cells implanted in mammary tissues) with those in a model of pulmonary metastases (E0771 cells injected intravenously). Tumor immune cell composition and TAM phenotypes were examined 1.5 and 3 weeks after tumor cell injection. Alveolar macrophages were excluded from the analysis of lung tumors. In general, the pattern of macrophage accumulation in model metastases was similar to that seen in model primary tumors, although this accumulation was slightly delayed in the metastases. Relative to primary tumors, metastases displayed a decreased accumulation of inflammatory monocytes, but displayed a similar robust accumulation of neutrophils (Fig 7A). The phenotype of TAMs in the E0771 model of metastases was very similar to that seen in primary tumors, with a predominance of CD11b^+^ MHC class II^-^ macrophages, the majority of which were Ly6C^+^ (Fig 7B). At both time points examined, primary tumors and metastases displayed virtually identical expression of MerTK and CCR2 on all TAM subsets (Fig 7C). These findings suggest that tumor immune cell composition and TAM phenotypes are determined primarily by the specific tumor cell type, irrespective of the tissue in which the tumor is located.

## Discussion

In this paper, we describe a protocol for performing flow cytometric analysis that allows the accurate identification and quantification of all immune cell types in almost any non-lymphoid tissues. To our knowledge, this is the first description of a one-panel flow cytometry protocol that is both comprehensive and widely applicable. The protocol we describe, including the tissue preparation method, basic staining protocol, and gating strategy, offers several advantages over previously described methods of tissue immunophenotyping. It minimizes cell loss due to cell fractionation or cell death and allows the accurate quantification of all major immune cell types in all tissues we have examined. It unambiguously distinguishes macrophages from dendritic cells and allows the identification and quantification of cell types, such as pulmonary interstitial macrophages, that have previously been difficult to analyze as distinct populations. It is applicable to both normal tissues and tissues undergoing an inflammatory response. Finally, it can be easily expanded by the inclusion of additional antibodies to identify all immune cell types in a given tissue or provide detailed phenotyping of individual immune cell types. As an example, with the addition of CD103, subpopulations of dendritic cells in H1N1 and LPS lungs and other non-lymphoid organs can be examined and quantified (S3B–S3D, S4B–S4D and S7 Figs). Herein, we have provided a detailed description of this protocol and demonstrated its utility by making several observations about specific cell markers and cell types.

In our basic staining protocol, a panel of 9 color-conjugated antibodies plus a viability dye is used to identify all major immune cell types (S2 Table). We should note that this protocol requires the use of a 4-laser flow cytometer. In developing this protocol, we combined several individual strategies that have previously been shown to be advantageous. The use of CD45 and a Live/Dead exclusion dye allows the elimination of gradient purification and clearly distinguishes live immune cells from non-immune cells and dead cells. CD64 and/or CD24 expression has previously been shown to distinguish macrophages from DC in lungs, peritoneum, and intestines [14, 16]. Here we demonstrate that examination of CD64 vs, CD24 expression distinguishes these cell types in a wide variety of non-lymphoid tissues. We should point out however, that several controversies exist regarding the definition of macrophages *vs*. dendritic cells among specific cell types. First, skin Langerhans cells are CD64^-^ and have traditionally been classified as dendritic cells. However, recent lineage tracing studies reveal that skin Langerhans cells are derived from yolk sac macrophages and fetal monocytes and fetal origin [33]. Using this ontologic definition, skin Langerhans cells would be classified as macrophages. Because the majority of the literature has classified these cells as dendritic cells, we have grouped them with CD64^-^CD24^-^ dendritic cells here. Second, a recent study suggests that kidney CD64^+^ cells arise from DNGR-1-expressing common dendritic cell precursors and should therefore be classified as DCs [17]. However, evidence that kidney CD64^+^ cells represent macrophages also exists. This remains area of contention [34]. Here we classify these cells as macrophages with the caveat that kidney CD64^+^ cells may represent a special case.

In terms of our gating strategy, it should be noted that once CD64^+^ macrophages are identified, additional markers and the testing of various gating strategies may be required to optimally distinguish macrophage subpopulations within each tissue and under different inflammatory conditions. Additionally, CD24 is expressed at high levels by both B cells and neutrophils, so it should only be used to identify DC after these cell types have been excluded using other markers. In addition, it is known that some DC subtypes, such as dermal DC, express little CD24 [21]. Thus, in cases where a defined population of CD64^-^CD24^-^ cells are found, further identification of the cell identity should be undertaken. For this reason, it is likely that gating will need to be adjusted in some tissues to a greater extent than we have demonstrated here. Ly6C is used routinely to distinguish the two major monocyte subsets, Ly6C^+^ inflammatory monocytes and Ly6C^-^ resident monocytes and to identify cells recently derived from inflammatory monocytes [35, 36]. Consistent with this, we find that Ly6C effectively distinguishes monocyte subsets in all tissues examined and is not expressed by any macrophage or classic DC population in normal tissues. However, we do find that Ly6C is expressed on ExMФ and some tumor associated macrophage populations. For Ly6C expressing TAM, it remains to be determined to whether this expression is due to their recent differentiation from monocytes or to the development of a unique tumor-specific phenotype.

To demonstrate the utility of our protocol, we quantified all major immune cell types in eight different normal tissues. We find that in non-mucosal organs, such as heart, liver, and kidney, immune cell composition is remarkably uniform, with each organ containing 25–30% lymphoid cells and 53–63% macrophages. Mucosal organs such as lungs and intestines contain a higher proportion of lymphoid cells (60–65%) and a lower proportion of macrophages (~20–30%). Among visceral organs, lungs contain the highest proportion of both neutrophils and dendritic cells. This may represent the constant exposure of lungs to environmental immune stimuli. As expected, skin contains large resident populations of Langerhans cells, dermal DC, and mast cells, while in eyes microglia and macrophages represent the majority (70%) of resident immune cells. Mammary glands are unique in that they contain a large number of eosinophils (25% of total immune cells). This is likely due to the requirement for eosinophils in postnatal mammary gland development [37].

In the course of validating our staining and gating strategy, we examined a number of different markers that have been used previously to identify macrophages or specific macrophage subsets. Consistent with previous reports, we find both MerTK and CD169 to be highly specific macrophage markers [16]. However, the expression levels of MerTK and CD169 are significantly lower than that of CD64, making them less useful as a primary means to identify macrophages via flow cytometry. CD68 is used primarily in histological staining to identify macrophages, but has been used for this purpose in some flow cytometric studies. Because CD68 protein is localized within cytoplasmic granules rather than on the cell surface [38], it would not be expected to be particularly useful for flow cytometric analysis [9]. Consistent with this, we find only low levels of CD68 surface expression on most macrophage populations. CD206 is expressed on macrophages and some DC populations. It is considered to be a marker of alternatively activated (M2) macrophages due to the fact that its expression is increased on macrophages stimulated with IL-4 and decreased on macrophages stimulated with IFN-γ [39, 40]. Unfortunately, some have interpreted this as CD206 being a specific marker for M2 macrophages, which is not the case, as CD206 is known to be expressed on multiple resident macrophage populations [23]. We find that CD206 is expressed at high levels on macrophages in all normal tissues examined. For this reason, it is unlikely to be useful in the identification of M2 macrophages unless changes in CD206 expression are examined. It is also important to note that F4/80, CD14, Mac2, and Mac3 expression are not limited to macrophages (S1 Table).

Similar to the non-specific macrophage markers discussed above, we find that the examination of GFP expression in *Cx3cr1*^*wt/GFP*^ mice cannot be used to specifically identify either macrophages or dendritic cells in non-lymphoid organs. Although, as previously described, we find monocytes to be uniformly GFP^+^ in *Cx3cr1*^*wt/GFP*^ mice [24], the expression of this marker in macrophages varies significantly by tissue. In lungs, intestines, and kidneys, the vast majority of resident or interstitial macrophages are GFP^+^; however, alveolar, heart, liver, and skin macrophages are predominately GFP^-^. In eyes, microglia are GFP^+^, but tissue macrophages are GFP^-^. Among dendritic cell populations, we find CD11b^-^ DC to be GFP^-^ and GFP expression by CD11b^+^ DC to be highly variable. A complete listing of the expression pattern of myeloid markers on specific cells types in each tissue is presented in S1 Table.

We also find that our basic staining protocol has considerable utility in the examination of inflamed tissues and tumors. Examination of inflammatory responses to LPS exposure and H1N1 infection are presented here. In addition, we have used this protocol to examine a wide variety of other acute and chronic inflammatory conditions in non-lymphoid organs and found it to be consistently reliable (data not shown). Additionally, this protocol allows quantification of all major immune cell types in tumors and the clear identification of macrophage subpopulations. Moreover, by examining the expression of three markers already included in our panel, CD11b, MHC class II, and Ly6C, we were able to identify several distinct TAM subsets. Further characterization of these subsets was accomplished by the inclusion of a few additional markers to our basic panel. Although our analysis was in no way definitive, we were able to make the observations that TAM phenotypes appear to arise in a highly tumor-specific manner and that these phenotypes tend to be preserved between model primary tumors and distal site metastases. It is possible that, if applied to humans, a similar TAM phenotyping strategy would yield important prognostic information.

In conclusion, we have described a protocol for the flow cytometric analysis of non-lymphoid tissues that allows the simultaneous identification and quantitation of all major leukocyte populations. This protocol is applicable to both normal and abnormal tissues, can be used to isolate individual cell types by fluorescence-activated cell sorting, and can be readily expanded by the inclusion of additional antibodies to permitting a detailed characterization of specific cell populations. We believe that this protocol will provide a valuable tool to examine immune cell repertoires and follow immune responses in a wide variety of tissues and experimental conditions.

## Supporting Information

S1 Fig Flow cytometric analysis of mouse lungs using extended antibody panel to identify mast cells and basophils. **A.** Contour plots and gating strategy used for the identification of major immune cell populations plus mast cells and basophils in normal mouse lungs. Gates containing multiple cell populations are numbered (R1-R11). Gates containing a single cell population are labeled with the included cell type. For this study, anti-FcɛR1 and CD117 (ckit) antibodies were added to our basic staining panel. Due to the variable expression of CD11b on basophils in various tissues, the R6 gate was extended to include CD11b^int^ cells, the majority of which are NK cells. There are exceedingly small numbers of mast cells and basophils in the lung. Basophils are more easily found in the blood (data not shown). Mast cells are more easily found in the trachea and skin (data not shown). **B.** Histogram plots of NK cells, B cells, and T cells-specific markers to confirm cellular identity. **C.** Contour plots showing alternative gating or R9, which contain DC and eosinophils.
(TIF)

S2 Fig Flow cytometric analysis of H1N1 infected lung tissue. Contour plots of windows and gating strategy used for the identification of major immune cell populations in Day 7 H1N1 infected lung tissue. Gates containing multiple cell populations are numbered (R1-R9). Gates containing a single cell population are labeled with the included cell type. Subset identification and more detailed phenotyping of CD64^+^ cells within the macrophage (MФ) gate.
(TIF)

S3 Fig Additional flow cytometry analysis of H1N1 infection. **A.** Dot plots of windows demonstrating various gating strategy for pulmonary macrophages in H1N1 infection. **B.** Dot plots of windows and gating strategy for identification of dendritic cell subsets. **C.** Histogram analyses of macrophage-specific and macrophage-associated markers in various cell type of MPS. **D.** Myeloid cells as percentage of CD45+ cells.
(TIF)

S4 Fig Additional flow cytometry analysis of LPS exposed lung tissues. **A.** Dot plots of windows demonstrating various gating strategy for pulmonary macrophages in LPS exposure. **B.** Dot plots of windows and gating strategy for identification of dendritic cell subsets. **C.** Histogram analyses of macrophage-specific and macrophage-associated markers in various cell type of MPS. **D.** Myeloid cells as percentage of CD45+ cells.
(TIF)

S5 Fig Flow cytometric analysis of normal mammary tissue. Contour plots of windows and gating strategy used for the identification of major immune cell populations in normal mouse mammary tissues. Gates containing multiple cell populations are numbered (R1-R9). Gates containing a single cell population are labeled with the included cell type. Subset identification and more detailed phenotyping of CD64^+^ cells within the macrophage (MФ) gate are shown in Fig 4C and 4D.
(TIF)

S6 Fig Flow cytometric analysis of MMTV-PyMT tumors. Contour plot of windows and gating strategy used for the identification of major immune cell populations in MMTV-PyMT tumors. Gates containing multiple cell populations are numbered (R1-R9). Gates containing a single cell population are labeled with the included cell type. Subset identification and more detailed phenotyping of CD64^+^ cells within the macrophage (MФ) gate are shown in Fig 6C and 6D.
(TIF)

S7 Fig Identification of dendritic cell subsets in non-lymphoid organs. Dendritic cells are further stained with CD103 further define dendritic cell subsets.
(TIF)

S1 Table Expression levels of commonly used myeloid cell markers on the major mononuclear phagocyte cell types found in individual tissues. (TIF)

S2 Table Antibodies and staining reagents used in the described studies. (TIF)
