# Supplementary figures and images for: A Protocol for the Comprehensive Flow Cytometric Analysis of Immune Cells in Normal and Inflamed Murine Non-Lymphoid Tissues
Source: PLoS One. 2016 Mar 3;11(3):e0150606. doi: 10.1371/journal.pone.0150606 (PMC4777539; doi:10.1371/journal.pone.0150606)

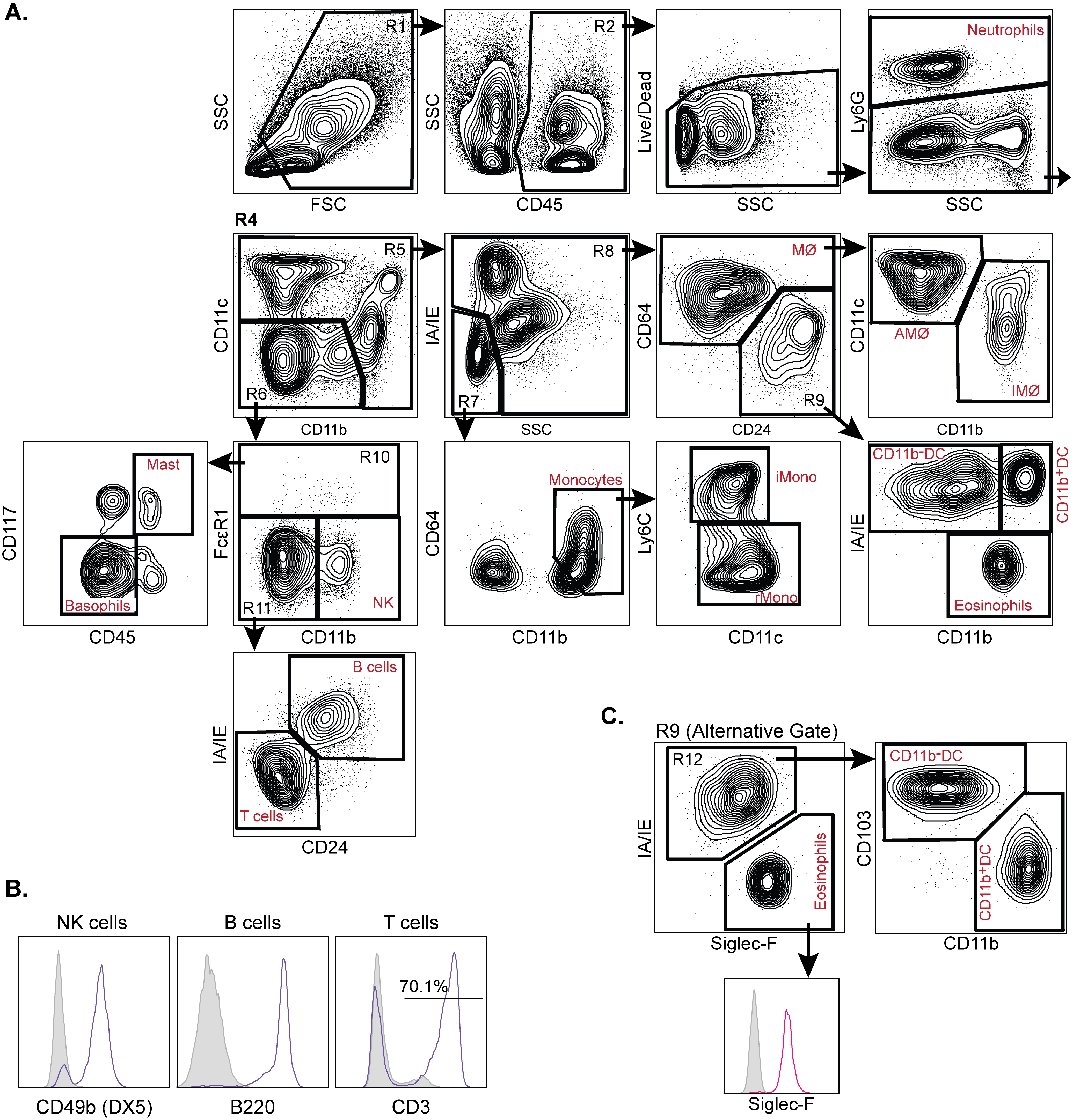

Supplement: S1 Fig — A. Contour plots and gating strategy used for the identification of major immune cell populations plus mast cells and basophils in normal mouse lungs. Gates containing multiple cell populations are numbered (R1-R11). Gates containing a single cell population are labeled with the included cell type. For this study, anti-FcɛR1 and CD117 (ckit) antibodies were added to our basic staining panel. Due to the variable expression of CD11b on basophils in various tissues, the R6 gate was extended to include CD11bint cells, the majority of which are NK cells. There are exceedingly small numbers of mast cells and basophils in the lung. Basophils are more easily found in the blood (data not shown). Mast cells are more easily found in the trachea and skin (data not shown). B. Histogram plots of NK cells, B cells, and T cells-specific markers to confirm cellular identity. C. Contour plots showing alternative gating or R9, which contain DC and eosinophils. (TIF) [file pone.0150606.s001.tif]

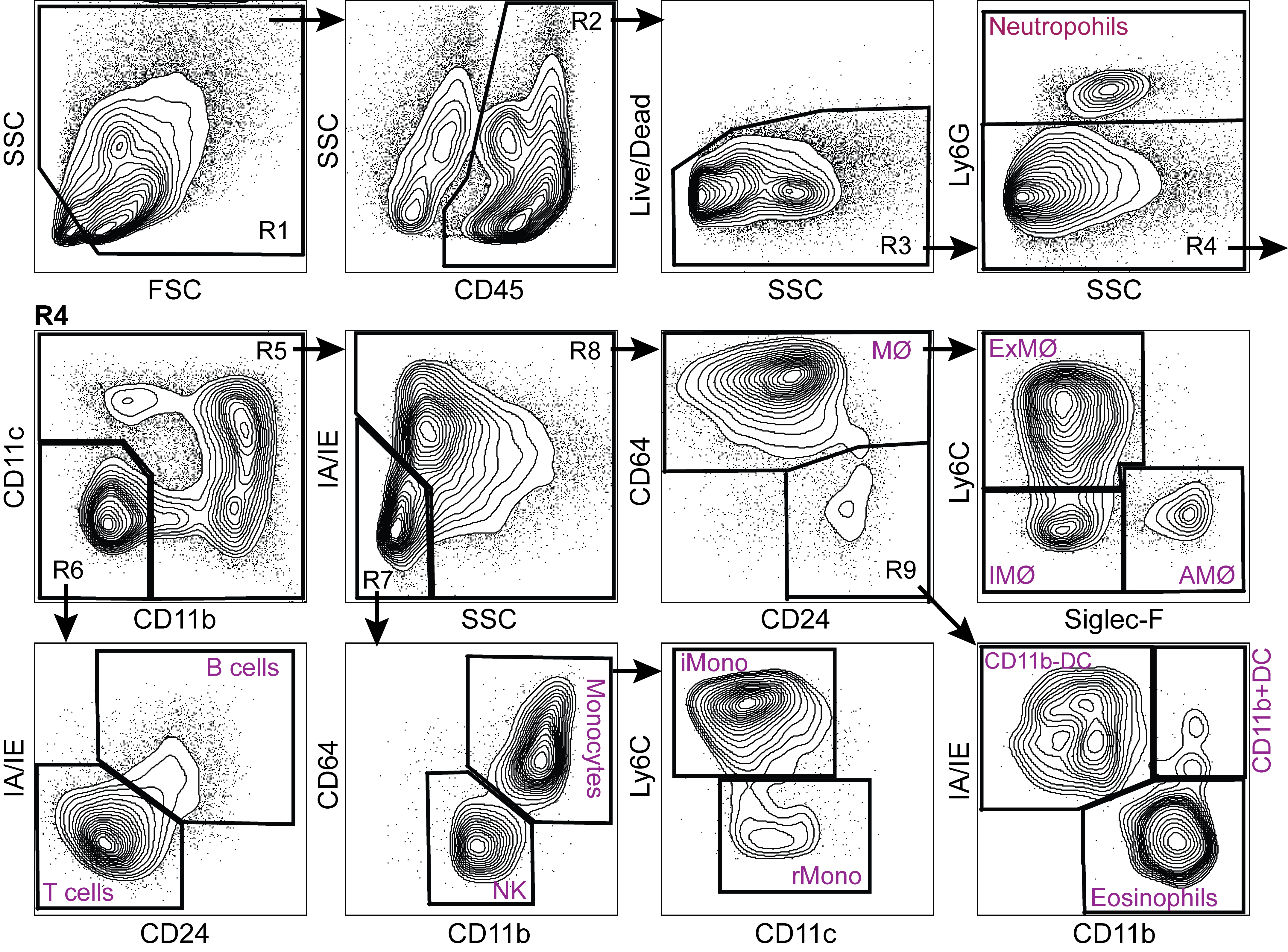

Supplement: S2 Fig — Contour plots of windows and gating strategy used for the identification of major immune cell populations in Day 7 H1N1 infected lung tissue. Gates containing multiple cell populations are numbered (R1-R9). Gates containing a single cell population are labeled with the included cell type. Subset identification and more detailed phenotyping of CD64+ cells within the macrophage (MФ) gate. (TIF) [file pone.0150606.s002.tif]

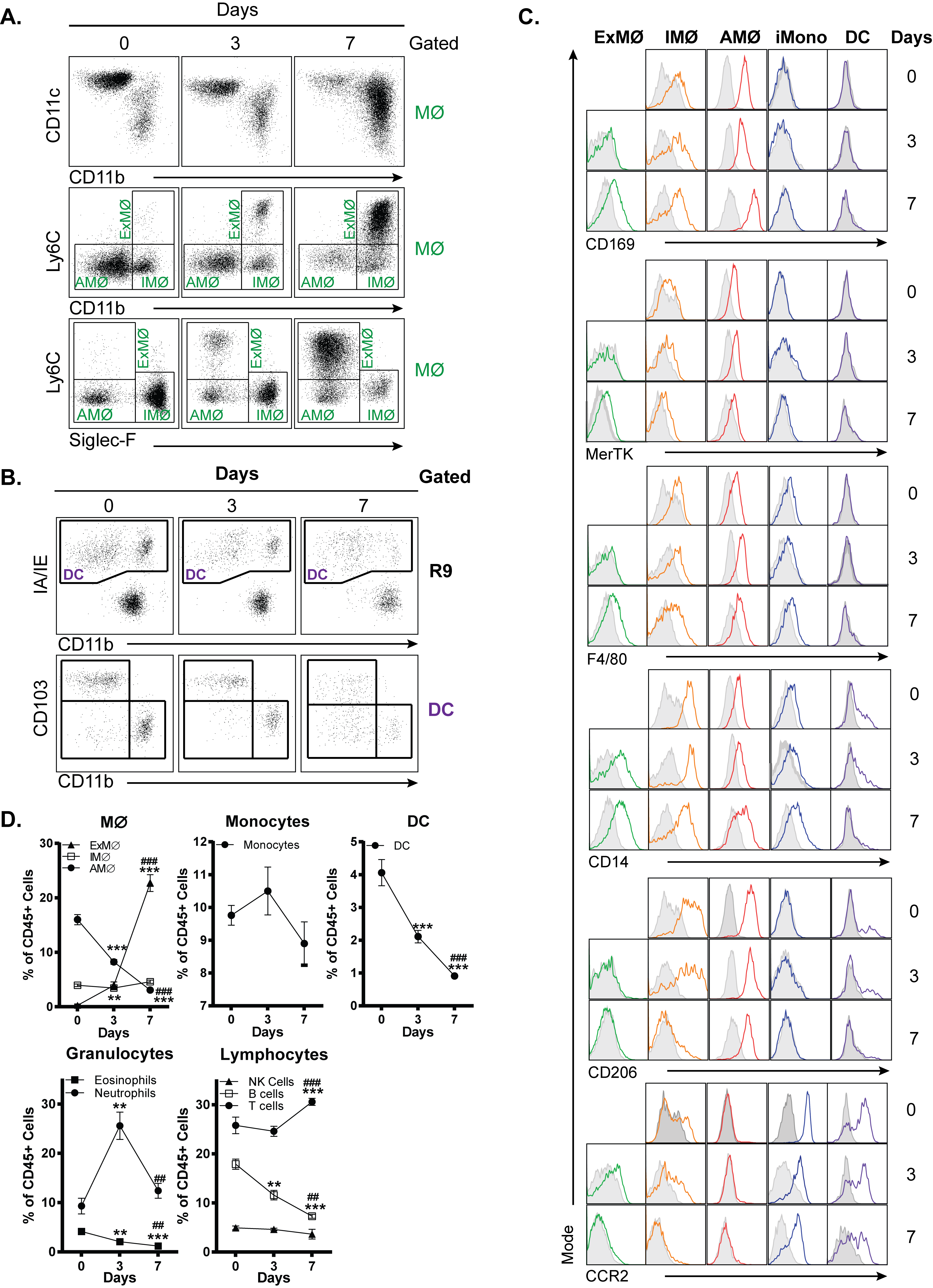

Supplement: S3 Fig — A. Dot plots of windows demonstrating various gating strategy for pulmonary macrophages in H1N1 infection. B. Dot plots of windows and gating strategy for identification of dendritic cell subsets. C. Histogram analyses of macrophage-specific and macrophage-associated markers in various cell type of MPS. D. Myeloid cells as percentage of CD45+ cells. (TIF) [file pone.0150606.s003.tif]

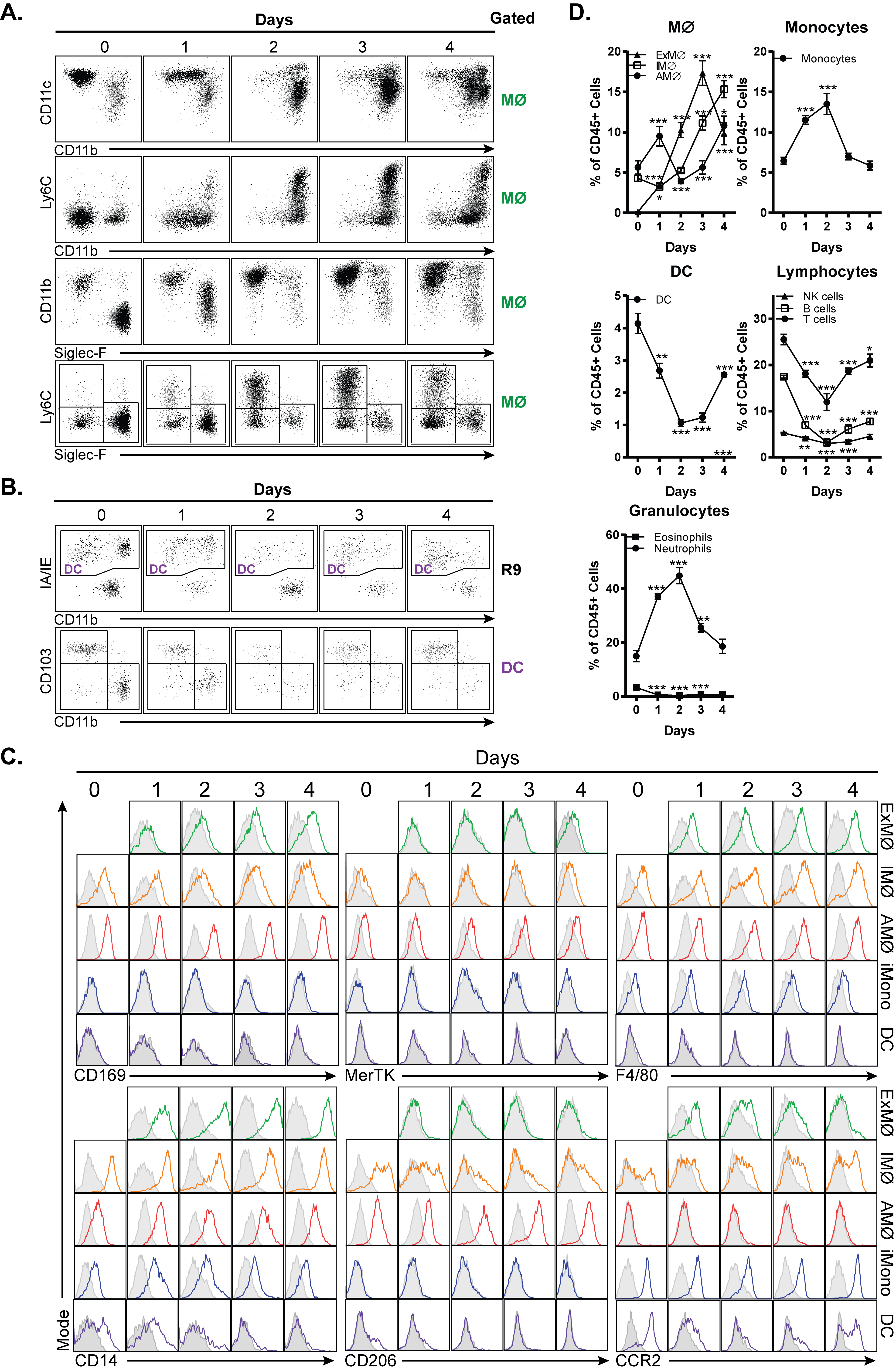

Supplement: S4 Fig — A. Dot plots of windows demonstrating various gating strategy for pulmonary macrophages in LPS exposure. B. Dot plots of windows and gating strategy for identification of dendritic cell subsets. C. Histogram analyses of macrophage-specific and macrophage-associated markers in various cell type of MPS. D. Myeloid cells as percentage of CD45+ cells. (TIF) [file pone.0150606.s004.tif]

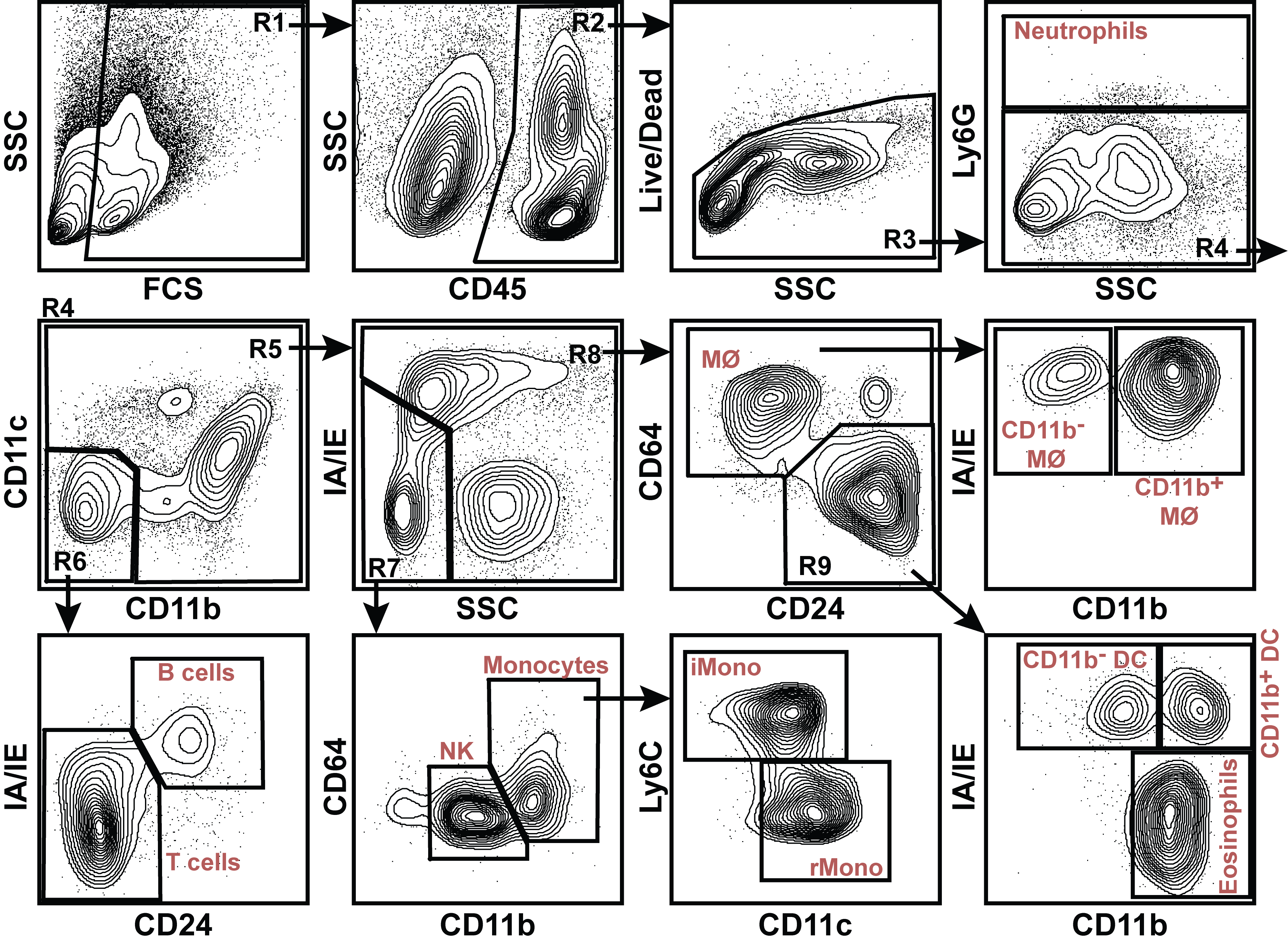

Supplement: S5 Fig — Contour plots of windows and gating strategy used for the identification of major immune cell populations in normal mouse mammary tissues. Gates containing multiple cell populations are numbered (R1-R9). Gates containing a single cell population are labeled with the included cell type. Subset identification and more detailed phenotyping of CD64+ cells within the macrophage (MФ) gate are shown in Fig 4C and 4D. (TIF) [file pone.0150606.s005.tif]

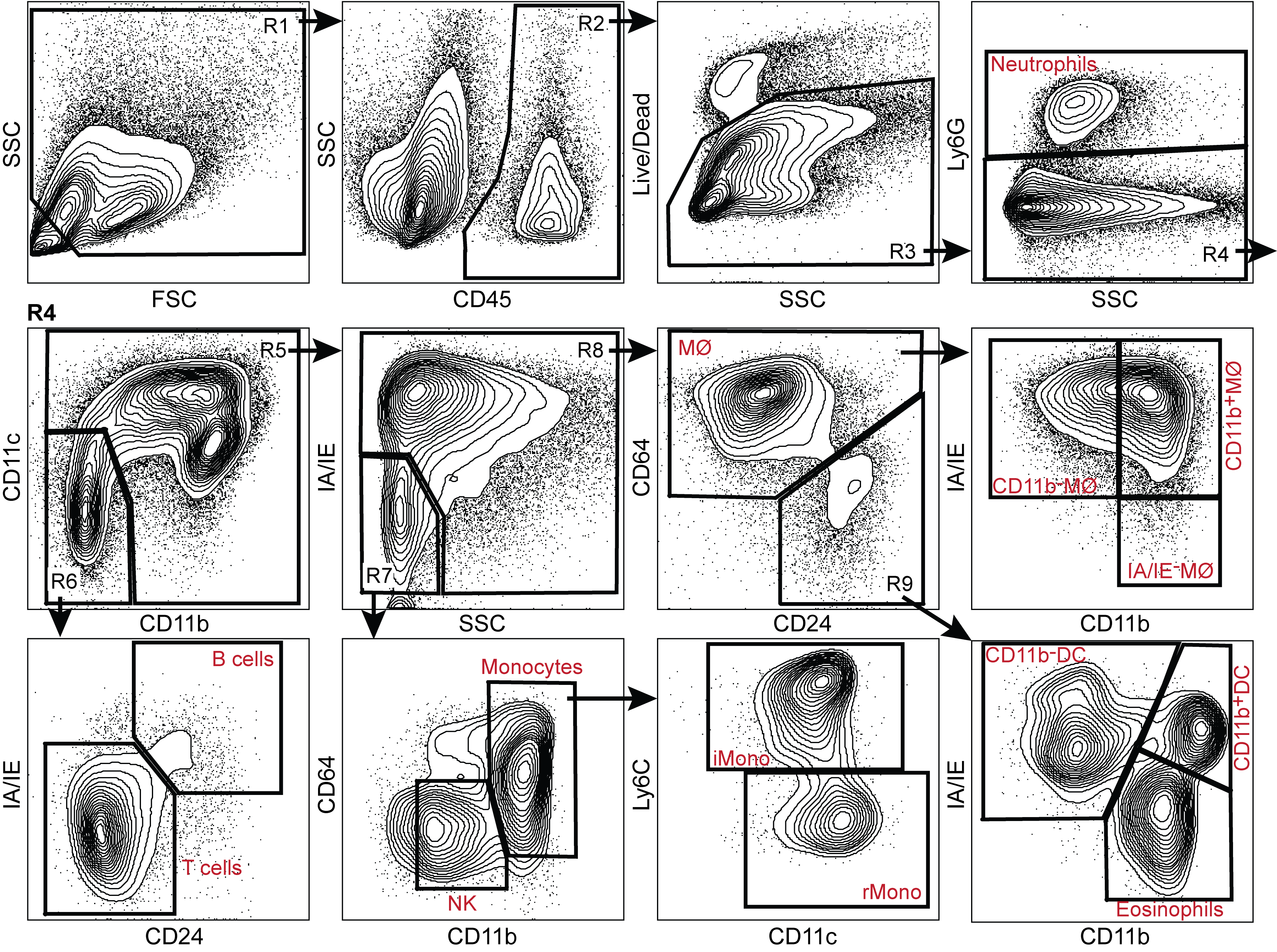

Supplement: S6 Fig — Contour plot of windows and gating strategy used for the identification of major immune cell populations in MMTV-PyMT tumors. Gates containing multiple cell populations are numbered (R1-R9). Gates containing a single cell population are labeled with the included cell type. Subset identification and more detailed phenotyping of CD64+ cells within the macrophage (MФ) gate are shown in Fig 6C and 6D. (TIF) [file pone.0150606.s006.tif]

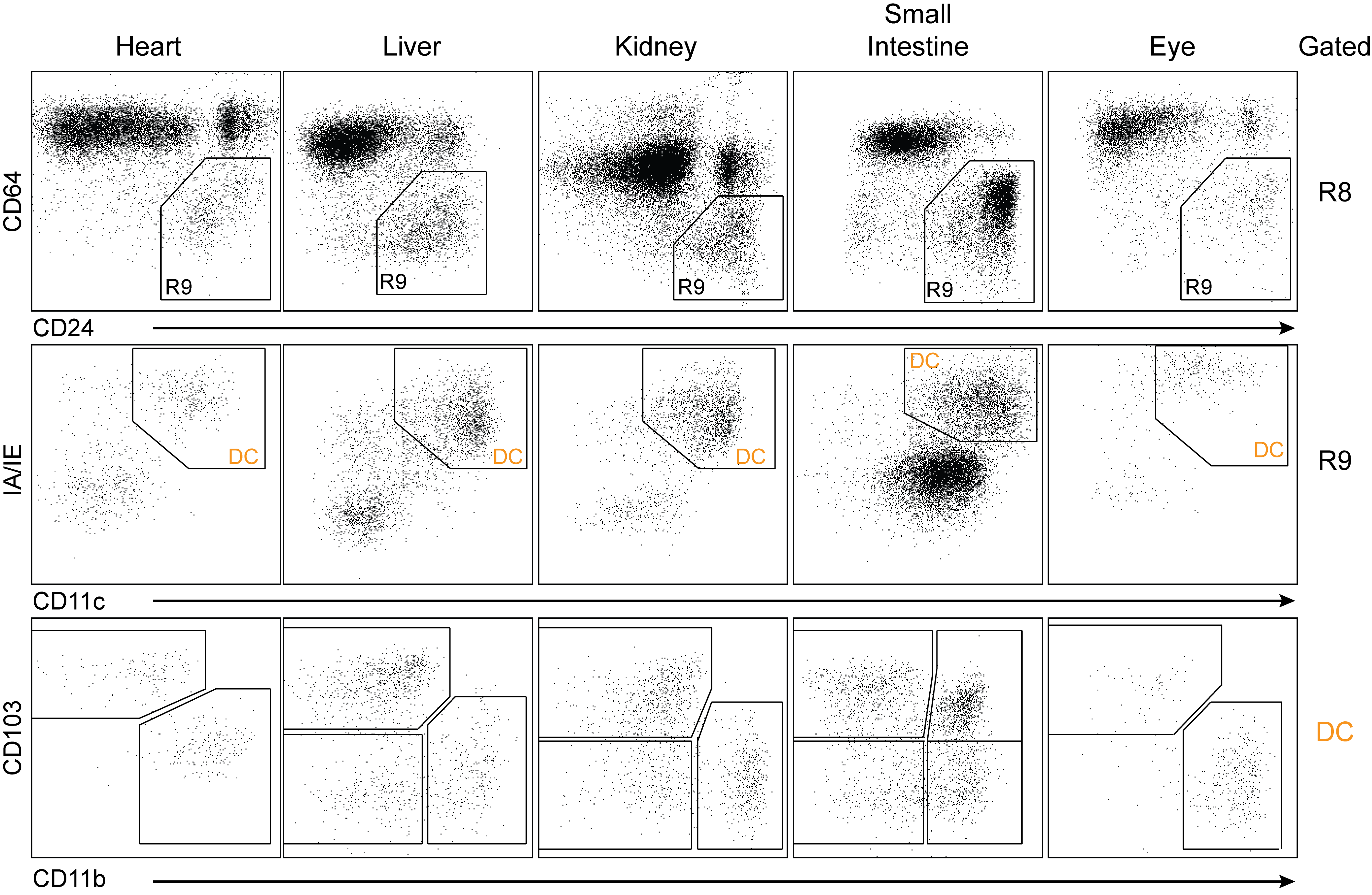

Supplement: S7 Fig — Dendritic cells are further stained with CD103 further define dendritic cell subsets. (TIF) [file pone.0150606.s007.tif]

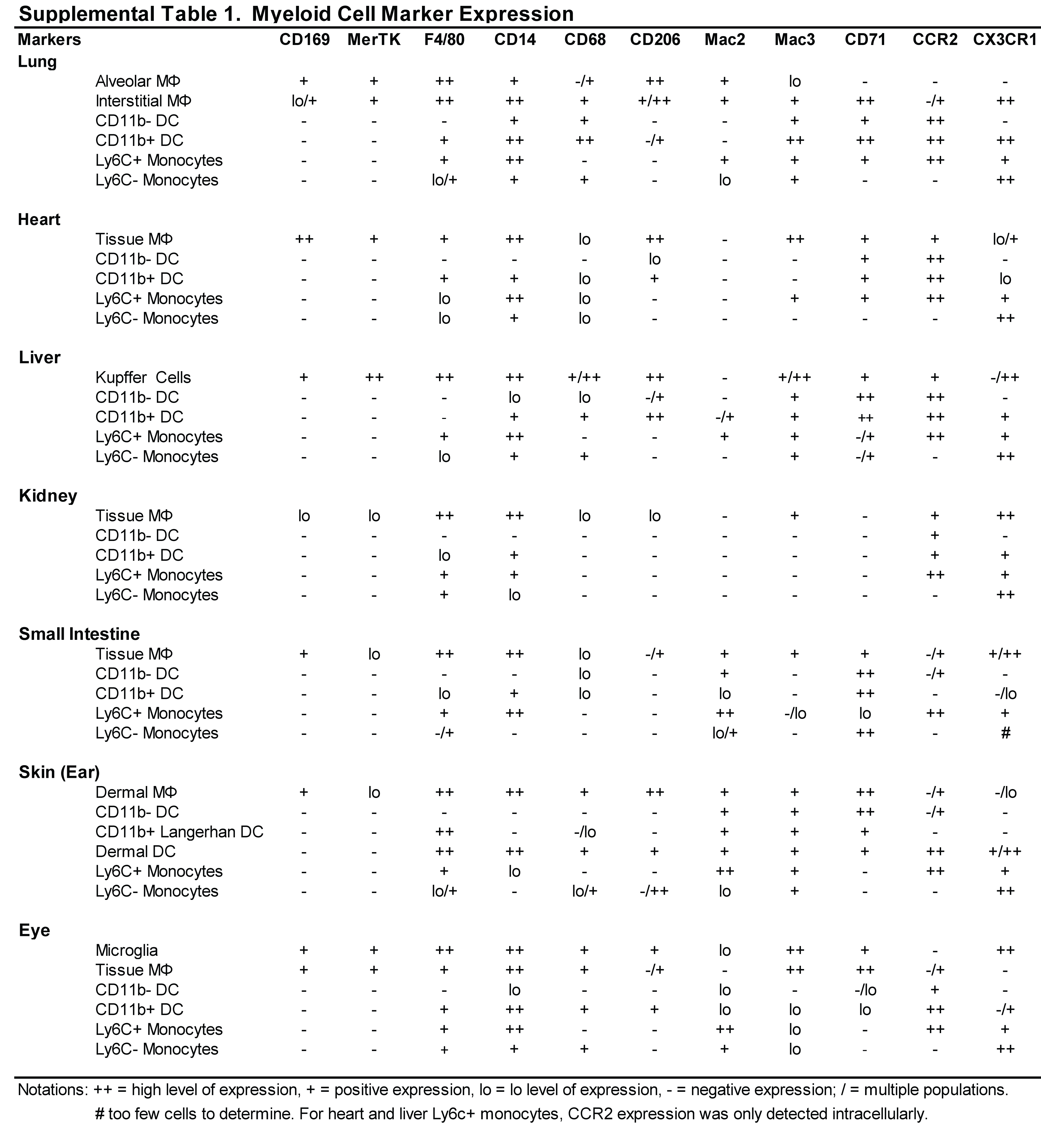

Supplement: S1 Table — (TIF) [file pone.0150606.s008.tif]

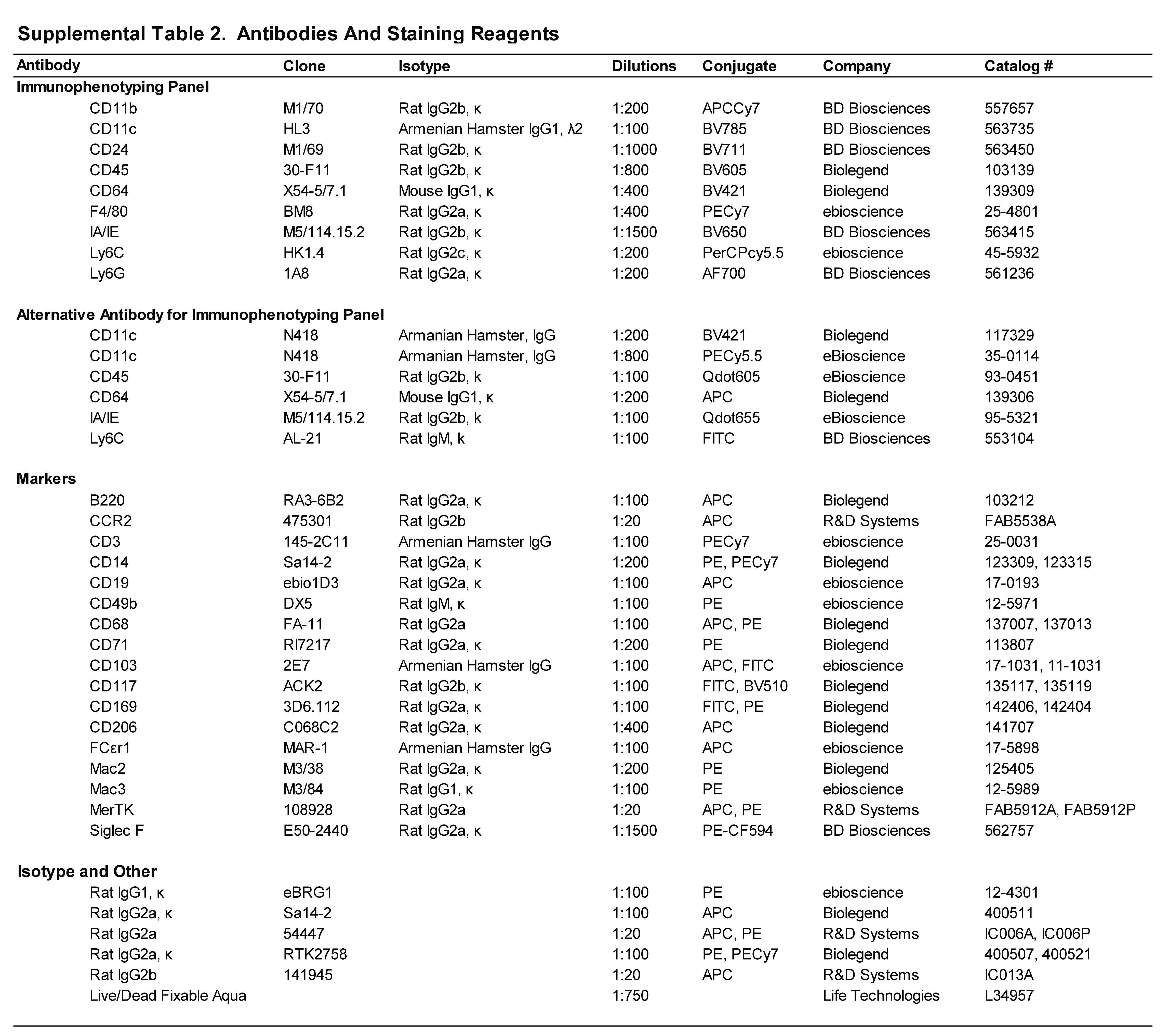

Supplement: S2 Table — (TIF) [file pone.0150606.s009.tif]
